# Red Yeast Rice for Hyperlipidemia: A Meta-Analysis of 15 High-Quality Randomized Controlled Trials

**DOI:** 10.3389/fphar.2021.819482

**Published:** 2022-01-17

**Authors:** Pengfan Li, Qi Wang, Kanjun Chen, Shihui Zou, Shi Shu, Chanchan Lu, Shiyun Wang, Yunqin Jiang, Chunxiang Fan, Yue Luo

**Affiliations:** ^1^ Department of Traditional Chinese Medicine, Punan Hospital, Shanghai, China; ^2^ Department of Traditional Chinese Medicine, Shuguang Hospital Affiliated to Shanghai University of Traditional Chinese Medicine, Shanghai, China; ^3^ Department of Encephalopathy, Guangyuan Hospital of Traditional Chinese Medicine, Guangyuan, China; ^4^ Shanghai Skin Disease Hospital, School of Medicine, Tongji University, Shanghai, China

**Keywords:** red yeast rice, hyperlipidemia, high-quality, RCTs, meta-analysis

## Abstract

**Background:** RYR is a commonly used lipid-lowering dietary supplements in Asian and European countries, showing considerable benefits and low toxicity. This quantitative study aims to present high-quality evidence regarding the efficacy and safety of RYR in treating hyperlipidemia, in order to promote its clinical application.

**Methods:** PubMed, embase, and Cochrane Central Register of Controlled Trials databases were systematically searched, and high-quality randomized controlled trials comparing RYR with non-RYR interventions were included. RevMan5.3 software was used to conduct the meta-analysis.

**Results:** A total of 1,012 individuals participated in this study (481 in the experimental and 531 in the control group). In comparison to statins, RYR was more effective in lowering TG (MD, −19.90; 95% CI, −32.22 to −7.58; *p* = 0.002), comparable in lowering LDL-C and elevating HDL-C, and less effective in lowering TC (MD, 12.24; 95% CI, 2.19 to 22.29; *p* = 0.02). Compared with nutraceutical, RYR significantly reduced TC (MD, −17.80; 95% CI, −27.12 to −8.48; *p* = 0.0002) and LDL-C (MD, −14.40; 95% CI, −22.71 to −6.09; *p* = 0.0007), and elevated HDL-C (MD, 7.60; 95% CI, 4.33 to 10.87; *p* < 0.00001). Moreover, RYR effectively synergized nutraceutical to further reduce TC (MD, −31.10; 95% CI, −38.83 to −23.36; *p* < 0.00001), LDL-C (MD, −27.91; 95% CI, −36.58 to −19.24; *p* < 0.00001), and TG (MD, −26.32; 95% CI, −34.05 to −18.59; *p* < 0.00001). Additionally, RYR significantly reduced apoB (MD, −27.98; 95% CI, −35.51 to −20.45; *p* < 0.00001) and, whether alone or in combination, did not increase the risk of adverse events in patients with hyperlipidemia.

**Conclusion:** RYR at 200–4800 mg daily appears to be a safe and effective treatment for hyperlipidemia, effectively regulating blood lipid levels with an exceptional impact on TG. Looking forward, high-quality clinical trials with longer observation periods are required to evaluate the efficacy and safety of RYR as a long-term medication.

**Systematic Review Registration**: (https://www.crd.york.ac.uk/PROSPERO/), identifier (CRD4202128450).

## Introduction

Hyperlipidemia is a multifactorial disease related to genetics and environment, which mainly manifests as plasma dyslipidemia. This includes increased total cholesterol (TC), low-density lipoprotein cholesterol (LDL-C), and triglycerides (TG), as well as reduced high-density lipoprotein cholesterol (HDL-C) (Tietge, 2014). Hyperlipidemia has been shown to significantly increase the risk of atherosclerosis, stroke, myocardial infarction, and other cardiovascular and cerebrovascular diseases (CVD) with patients being twice as likely to suffer from CVD as compared to normolipidemic individuals (Wood, 2001; Karr, 2017). The global annual deaths due to CVD was 17.0 million in 2010s, and it is estimated to reach 24.8 million in 2030 (Estruch et al., 2018). It is well known that an increase in LDL-C concentration is a risk factor for CVD and is therefore considered to be the primary goal of CVD prevention and treatment (Jung et al., 2014). Currently, statins are the most effective class of drugs for improving lipid profiles, in turn helping to reduce the risk of CVD (Collins et al., 2003; Sever et al., 2003).

Although statins are generally well tolerated, a minority of patients experience side effects that reduce treatment adherence, limiting the full potential of the drug for risk reduction (Bates et al., 2009). In clinical practice, the prevalence of statin intolerance may be as high as 10%, with the risk factors for statin intolerance including older age, female sex, renal disease, history of muscle symptoms, and high statin dose (Bruckert et al., 2005). Since these risk factors are often exclusion criteria for clinical trials, the prevalence of statin intolerance in trial populations is lower than expected in comparison to in clinical practice (Stroes et al., 2015). Therefore, as an alternative to statins, certain ingredients from foods which have mild or no side effects have gained increasing attention for use in the treatment of hyperlipidemia.

RYR, a traditional Chinese medicine fermented from rice grain by *Monascus purpureus Went.*, is well known for its beneficial effects on cardiovascular and cerebrovascular health (Kalaivani et al., 2010). Currently, it is one of the most commonly used lipid-lowering dietary supplements in Asian and European countries (Sahebkar et al., 2016). Through fermentation, *Monascus purpureus Went.* enriches rice with complex substances, such as monacolin and other polyketides, that show important lipid-lowering activity (Ma et al., 2000). Depending on the specific fermentation conditions and yeast strain used, several types of monacolin (e.g., monacolin m, l, J, x, and compactin) have been identified. The subtype of monacolin K is of specific interest, as it is structurally identical to lovastatin (Alberts, 1988).

In recent years, although a few systematic reviews have been published on the benefits of RYR with regard to blood lipid distribution and cardiovascular and cerebrovascular diseases, most have been of low quality (Gerards et al., 2015; Sungthong et al., 2020). Xuezhikang, a Chinese patent medicine with RYR as its primary component, has been listed in the primary prevention guidelines of cardiovascular diseases in China (Chinese Society of Cardiology, 2020). However, after the publication of case reports claiming it to be toxic, concerns were raised about the effectiveness and safety of RYR (Polsani et al., 2008; Russo et al., 2016; Raschi et al., 2018). Since then, a substantial amount of new data has been published. Therefore, the purpose of this quantitative study is to collect and present the most up to date evidence on the efficacy and safety of RYR in the treatment of hyperlipidemia.

## Methods

This study has been registered with PROSPERO (CRD42021284502). It is based on the Cochrane Handbook for Systematic Reviews of Interventions (Higgins and Green, 2008) and was proposed on the basis of the (PRISMA) guidelines for systematic review and meta-analysis (Page et al., 2021) (Supplementary Table S1).

### Search Strategy

A comprehensive literature search was conducted using Cochrane Library, PubMed, and Excerpta Medica database (embase). All databases were searched from their inception up until September 2021. We combined medical subject words (MeSH) and free text words to retrieve all possible studies, and MeSH terms were modified according to the specifications of each database. The detailed search strategy can be seen in Supplementary Table S2. Additionally, we searched the Chinese Clinical Trial Registry (http://www.chictr.org.cn/index.aspx) and Clinical Trials (http://www.clinicaltrials.gov) websites to identify protocols of high quality randomized controlled trials (RCTs).

### Inclusion and Exclusion Criteria

The inclusion criteria were as follows: 1) participants: patients diagnosed with hyperlipidemia; 2) intervention: red yeast rice alone or combined therapies; 3) comparison: conventional or placebo therapies; 4) outcomes: low-density lipoprotein cholesterol, total cholesterol, triglyceride, high density lipoprotein cholesterol, apolipoprotein A-I, apolipoprotein B, adverse events, and; 5) study design: randomized controlled trials. Studies were excluded if they met the following exclusion criteria: 1) participants with comorbidities; 2) different drug forms used in the experimental and control group; 3) co-interventions that used lipid-lowering drugs other than RYR, and; 4) not high-quality RCTs with a Jadad score ≥4 in efficacy and safety analysis, so that the efficacy and safety of RYR could not be judged.

### Data Extraction

All articles were strictly screened by two independent investigators (Q. Wang and S. Zou) according to the predetermined inclusion criteria. Two reviewers (P. Li and Q. Wang) completed the self-designed data extraction form which included general information (the first author, year, and baseline characteristics of patients), sample size, diagnostic criteria, interventions and control treatments, course of treatments, outcomes, and adverse reactions.

### Outcome Measures

The primary outcome for this study was the level of low-density lipoprotein cholesterol after treatment, which is a significant indicator of overall blood lipid levels, and can be utilized as a risk factor for cardiovascular and cerebrovascular diseases (Chen et al., 2012). The secondary outcomes included total cholesterol, triglyceride, high density lipoprotein cholesterol, apolipoprotein A-I, and apolipoprotein B levels, as well as adverse events.

### Risk of Bias in Individual Studies

For each included study, two investigators (P. Li and S. Shu) completed the Jadad scale used specifically to assess the quality of the evaluation method. A third-party (C. Lu) was consulted to resolve any disagreement between the two investigators. Four dimensions of the Jadad scale (total 7 points) were applied in this research, namely randomization, concealment, blind method, and reports of withdrawals and dropouts. Trials scoring 1–3 points were considered low quality and those that scored 4–7 considered as high.

### Statistical Analysis

RevMan5.3 software was used for data analysis. Risk ratios (RR) with 95% confidence intervals (CI) were evaluated for dichotomous data, and for continuous data, mean difference (MD) and standard mean difference (SMD) were used. Heterogeneity was analyzed using the I^2^ statistical test and the fixed effects model was used when there was homogeneity (I^2^<50%). Otherwise, a random effects model was applied. Subgroups analyses were performed to avoid heterogeneity and sensitivity analysis was used to assess bias. For all analyses, *p* values less than 0.05 were considered to be of statistical significance.

## Results

### Included Studies

**FIGURE 1:**
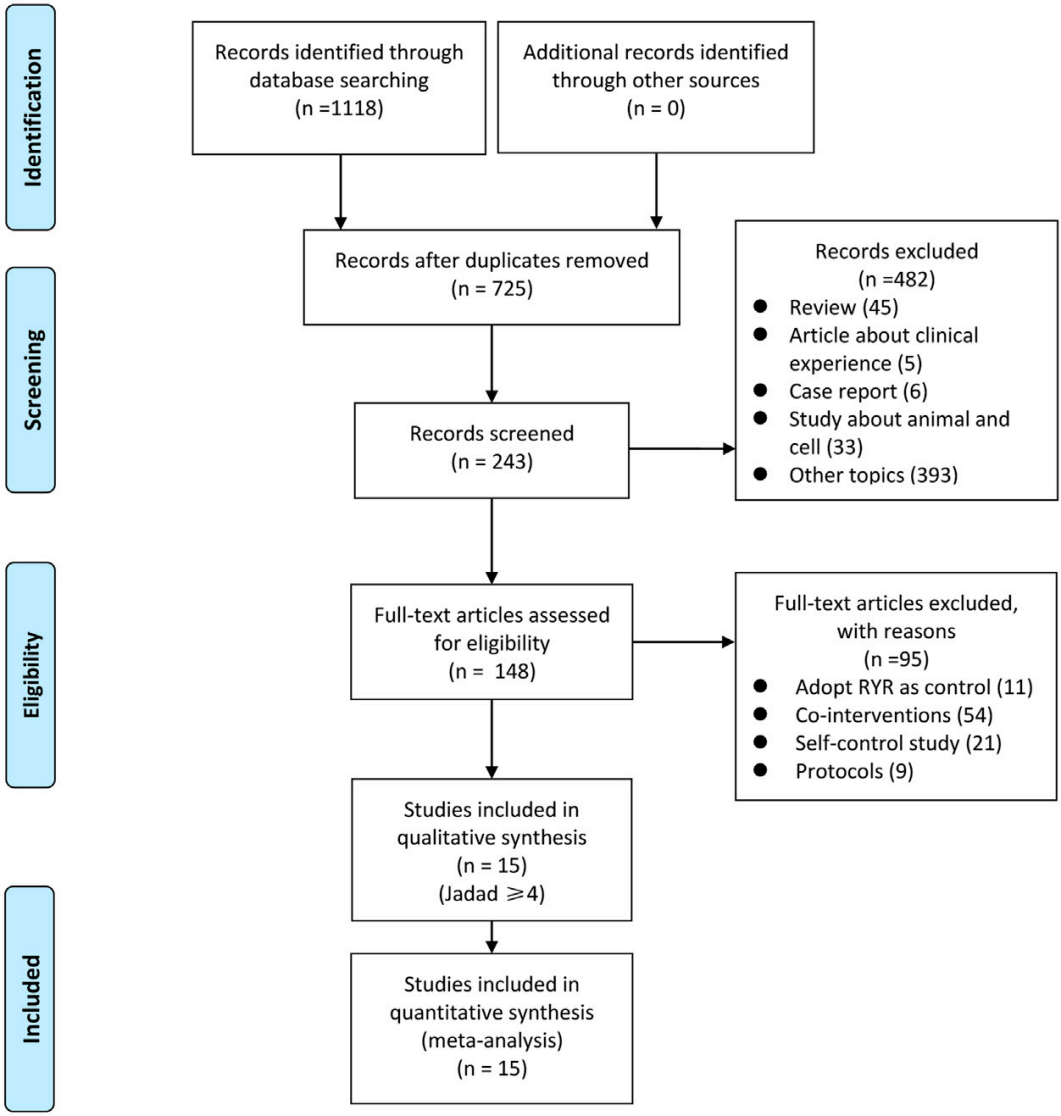
Flowchart of search strategy and study selection, according to the Preferred Reporting Items for Systematic Reviews and Meta-analyses (PRISMA) guidelines.

We identified 1,118 articles after a preliminary search of 3 databases. From these, 393 repetitive articles were excluded, and 482 articles were deleted upon screening the titles and abstracts. Among the remaining 243 studies, 95 were excluded for the following reasons: 11 adopted RYR in the control group; 54 used co-interventions other than RYR so that the efficacy and safety of RYR could not be judged; 21 were self-control studies, and; 9 articles were protocols of RCTs. Finally, the remaining 148 studies were evaluated with reference to the Jadad scores, and 15 of them were considered high quality (Jadad scale ≥4 points), meeting all inclusion criteria (Heber et al., 1999; Zhao et al., 2003; Lin et al., 2005; Becker et al., 2009; Yang et al., 2009; Bogsrud et al., 2010; Halbert et al., 2010; Karl et al., 2012; Cicero et al., 2013, Verhoeven et al., 2013; Moriarty et al., 2014; Cui et al., 2015; Wang et al., 2015; Heinz et al., 2016; Cicero et al., 2017). A flowchart which briefly summarizes the screening process can be seen in Figure 1.

### Study Characteristics

A total of 1,012 individuals were included in this study, with 481 in the experimental group and 531 in the control group. Eleven trials (Heber et al., 1999; Lin et al., 2005; Becker et al., 2009; Yang et al., 2009; Bogsrud et al., 2010; Halbert et al., 2010; Karl et al., 2012; Cicero et al., 2013, Verhoeven et al., 2013; Heinz et al., 2016; Cicerp et al., 2017) used RYR alone or in combination as the experimental group, and four (Zhao et al., 2003; Moriarty et al., 2014; Cui et al., 2015; Wang et al., 2015) used a compound named XZK (whose ingredient was RYR) alone as the experimental group. Regarding the control group, eleven trials (Heber et al., 1999; Zhao et al., 2003; Lin et al., 2005; Becker et al., 2009; Yang et al., 2009; Bogsrud et al., 2010; Karl et al., 2012; Cicero et al., 2013; Verhoeven et al., 2013; Moriarty et al., 2014; Heinz et al., 2016) used a placebo, three (Halbert et al., 2010; Cui et al., 2015; Wang et al., 2015) used statins, and three (Yang et al., 2009; Karl et al., 2012; Cicero et al., 2017) used nutraceuticals as control. Meanwhile, the treatment course for these studies ranged from 6 to 24 weeks.

LDL-C levels were recorded as the primary outcome in all 15 trials. All trials measured the main components of blood lipids (TC, TG, and HDL-C), with six trials (Zhao et al., 2003; Lin et al., 2005; Bogsrud et al., 2010; Cicero et al., 2013; Moriarty et al., 2014; Cui et al., 2015) also giving data on apoA-I and apoB. In terms of safety, adverse events were recorded in 13 trials (Heber et al., 1999; Lin et al., 2005; Becker et al., 2009; Yang et al., 2009; Bogsrud et al., 2010; Halbert et al., 2010; Karl et al., 2012; Cicero et al., 2013, Verhoeven et al., 2013; Moriarty et al., 2014; Wang et al., 2015; Heinz et al., 2016; Cicero et al., 2017). A summary table of the characteristics of all included trials is presented in Table 1.

**TABLE 1:** Summary of the characteristics of the included trials.

Author year	Sample size		Age (years) (Mean ± SD)		BMI(kg/m2) (Mean ± SD)		Course of treatment	Adverse events	
	E	C	E	C	E	C		E	C
Heber et al. (1999)	42	41	NR	NR	27 ± 6	27 ± 5	8w	1	3
Zhao et al. (2003)	25	25	57.9 ± 5.7	58.6 ± 5.7	24.8 ± 2.2	24.9 ± 1.6	6w	NR	NR
Lin et al. (2005)	39	40	46.3 ± 10.1	46.5 ± 9.5	24.3 ± 3.3	23.4 ± 2.7	8w	3	3
Becker et al. (2009)	30	29	NR	NR	28.8 ± 4.3	29.2 ± 5	24w	4	1
Yang et al. (2009)	18	18/10	54.4 ± 10.4	51.6 ± 8.6/56.3 ± 11.8	26.7 ± 3.5	24.7 ± 2.9/25.3 ± 3.1	6 m	0	0
Bogsrud et al. (2010)	22	20	NR	NR	NR	NR	16w	7	1
Halbert et al. (2010)	21	22	62.4 ± 8.9	62.9 ± 6.6	NR	NR	12w	16	16
Karl et al. (2012)	23	22/14	60 ± 13	58 ± 15/63 ± 9	NR	NR	8w	1	1
Cicero et al. (2013)	13	13	53.56 ± 8.76	51.28 ± 6.79	26.97 ± 0.92	26.62 ± 0.79	8w	0	0
Verhoeven et al. (2013)	31	21	55 ± 7	55 ± 11	NR	NR	8w	16	11
Moriarty et al. (2014)	42	36/38	56.3 ± 10.8	57.8 ± 9.0/56.0 ± 12.5	27.7 ± 3.9	26.2 ± 4.3/27.3 ± 3.8	12w	17	18
Cui et al. (2015)	30	30/30	67.04 ± 8.61	66.12 ± 9.11/65.36 ± 8.75	NR	NR	8w	1	0
Wang et al. (2015)	30	30	51.54 ± 12.70	51.54 ± 12.70	21.3 ± 1.5	22.7 ± 1.8	22w	8	2
Heinz et al. (2016)	70	72	57.5 ± 7.2	57.0 ± 6.8	26.9 ± 4.2	25.9 ± 3.9	12w	NR	NR
Cicero et al. (2017)	23/22	20	53 ± 11.2/49.9 ± 14.5	53.6 ± 12.3	25.6 ± 3.4/25.2 ± 2.9	25.0 ± 2.6	8w	0	0

Author year	Intervention		Outcome
	E	C	
Heber et al. (1999)	RYR (2400 mg daily)	Placebo	TC, TG, LDL-C, HDL-C, AEs
Zhao et al. (2003)	XZK (600 mg twice daily)	Placebo	TC, TG, LDL-C, HDL-C, apoA-I, apoB
Lin et al. (2005)	RYR (600 mg twice daily)	Placebo	TC, TG, LDL-C, HDL-C, apoA-I, apoB, AEs
Becker et al. (2009)	RYR (600 mg twice daily)	Placebo	TC, TG, LDL-C, HDL-C, AEs
Yang et al. (2009)	RYR (600 mg twice daily) + nattokinase (100 mg twice daily)	Nattokinase (100 mg twice daily)/Placebo	TC, TG, LDL-C, HDL-C, AEs
Bogsrud et al. (2010)	RYR (four capsules daily)	Placebo	TC, TG, LDL-C, HDL-C, apoA-I, apoB, AEs
Halbert et al. (2010)	RYR (2400 mg twice daily)	Pravastatin (40 mg daily)	TC, TG, LDL-C, HDL-C, AEs
Karl et al. (2012)	RYR (2400 mg daily) +Nutraceutical (15 ml daily)	Nutraceutical (15 ml daily)/Placebo	TC, TG, LDL-C, HDL-C, AEs
Cicero et al. (2013)	RYR (one tablet daily)	Placebo	TC, TG, LDL-C, HDL-C, apoA-I, apoB, AEs
Verhoeven et al. (2013)	RYR (two capsules daily)	Placebo	TC, TG, LDL-C, HDL-C, AEs
Moriarty et al. (2014)	XZK (1200 mg twice daily)	Placebo	TC, TG, LDL-C, HDL-C, apoA-I, apoB, AEs
Cui et al. (2015)	XZK (600 mg twice daily)	Simvastatin (20 mg daily) (after liver enzymes returned to normal)/simvastatin (20 mg daily) (continued)	TC, TG, LDL-C, HDL-C, apoA-I, apoB
Wang et al. (2015)	XZK (1200 mg daily) + rosuvastatin (5 mg daily)	Rosuvastatin (10 mg daily)	TC, TG, LDL-C, HDL-C, AEs
Heinz et al. (2016)	RYR (200 mg daily)	Placebo	TC, TG, LDL-C, HDL-C, AEs
Cicero et al. (2017)	RYR (one liquid stick daily) + Phytosterols (800 mg daily)/RYR (one liquid stick daily)	Phytosterols (800 mg daily)	TC, TG, LDL-C, HDL-C, AEs

E, experimental group; C, control group; RYR, red yeast rice; XZK, xue-zhi-kang capsule; TC, total cholesterol; TG, triglyceride; LDL-C, low density lipoprotein cholesterol; HDL-C, high density lipoprotein cholesterol; apoA-I, apolipoprotein A-I; apoB, apolipoprotein B; AEs, adverse events.

### Risk of Bias

The methodological quality of each trial was evaluated via Jadad score. Based on this assessment, all included trials were of high quality, having a Jadad score between 4 and 7. From the 15 included trials: eight (Heber et al., 1999; Zhao et al., 2003; Lin et al., 2005; Yang et al., 2009; Bogsrud et al., 2010; Cui et al., 2015; Wang et al., 2015; Heinz et al., 2016) only described the randomization method, making it unclear whether the specific method was appropriate; nine (Heber et al., 1999; Zhao et al., 2003; Lin et al., 2005; Yang et al., 2009; Bogsrud et al., 2010; Verhoeven et al., 2013; Cui et al., 2015; Wang et al., 2015; Heinz et al., 2016) did not detail the method of allocation concealment; six (Becker et al., 2009; Yang et al., 2009; Karl et al., 2012; Cicero et al., 2013; Cui et al., 2015; Wang et al., 2015) were described as double-blind, with no clear mention of the implementation method, and; one trial (Zhao et al., 2003) did not describe the methods of withdrawal and dropout. The funnel plot on the safety and efficacy of red yeast rice for hyperlipidemia was basically symmetric, suggesting no publication bias in the meta-analysis (Supplementary Figure S1). The remaining trials had a full Jadad score of 7 points. Further details regarding bias can be seen in Table 2.

**TABLE 2:** Jadad scale of the included trials.

Author year	JADAD				
	a	b	c	D	T
Heber et al. (1999)	1	1	2	1	5
Zhao et al. (2003)	1	1	2	0	4
Lin et al. (2005)	1	1	2	1	5
Becker et al. (2009)	2	2	1	1	6
Yang et al. (2009)	1	1	1	1	4
Bogsrud et al. (2010)	1	1	2	1	5
Halbert et al. (2010)	2	2	2	1	7
Karl et al. (2012)	2	2	1	1	6
Cicero et al. (2013)	2	2	1	1	6
Verhoeven et al. (2013)	2	1	2	1	6
Moriarty et al. (2014)	2	2	2	1	7
Cui et al. (2015)	1	1	1	1	4
Wang et al. (2015)	1	1	1	1	4
Heinz et al. (2016)	1	1	2	1	5
Cicero et al. (2017)	2	2	2	1	7

From a to d, dimension of the Jadad scale. Points awarded: a, study was described as randomized, 1 point; the method was appropriate (table of random numbers, computer generated, etc.), 2 points; b, study used allocation concealment, 1 point; the method was appropriate (taken by the third one who wasn’t researcher or patient, opaque envelope, etc.), 2 points; c, study was described as double blind, 1 point; the method was appropriate (identical placebo, active placebo, dummy, etc.), 2 points; d, study reported withdraws and dropouts and described the reasons. T, total.

### Primary Outcomes

#### Low Density Lipoprotein Cholesterol

**FIGURE 2:**
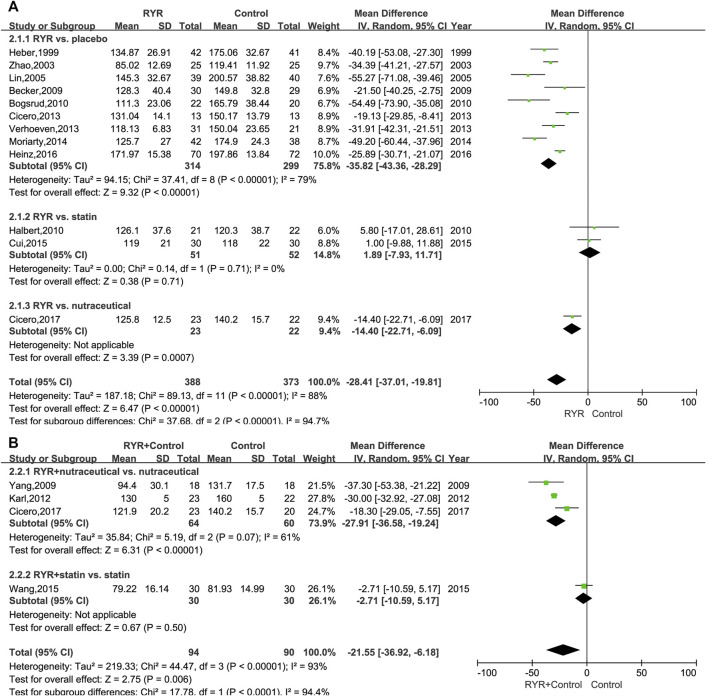
**(A)** Forest plot of LDL-C between RYR alone and control groups. **(B)** Forest plot of LDL-C between RYR in combination and control groups. (RYR: red yeast rice; LDL-C: low density lipoprotein cholesterol).

A comprehensive analysis of the LDL-C level data from each trial was conducted. The LDL-C level in patients with hyperlipidemia who were treated with RYR alone (MD, −28.41; 95% CI, −37.01 to −19.81; *p* < 0.00001; I^2^ = 88%; random effects model) or in combination (MD, −21.55; 95% CI, −36.92 to −6.18; *p* = 0.006; I^2^ = 93%; random effects model) was significantly lower than for patients in the control group. Additionally, we performed a subgroup analysis based on the different control group interventions. When compared with statins in the treatment of hyperlipidemia, RYR alone showed no significant difference (MD, 1.89; 95% CI, −7.93 to 11.71; *p* = 0.71). However, compared with nutraceutical and placebo control groups, the RYR group showed significantly reduced LDL-C levels (nutraceutical: MD, −14.40; 95% CI, −22.71 to −6.09; *p* = 0.0007; placebo: MD, −35.82; 95% CI, −43.36 to −28.29; *p* < 0.00001) (Figure 2A). When used in combination for treating hyperlipidemia, RYR and statins showed no significant difference compared with statins alone (MD, −2.71; 95% CI, −10.59 to 5.17; *p* = 0.50). However, patients treated with RYR in combination with a nutraceutical showed significantly reduced levels of LDL-C compared with nutraceuticals alone (MD, −27.91; 95% CI, −36.58 to −19.24; *p* < 0.00001) (Figure 2B).

### Secondary Outcome

#### Total Cholesterol

**FIGURE 3:**
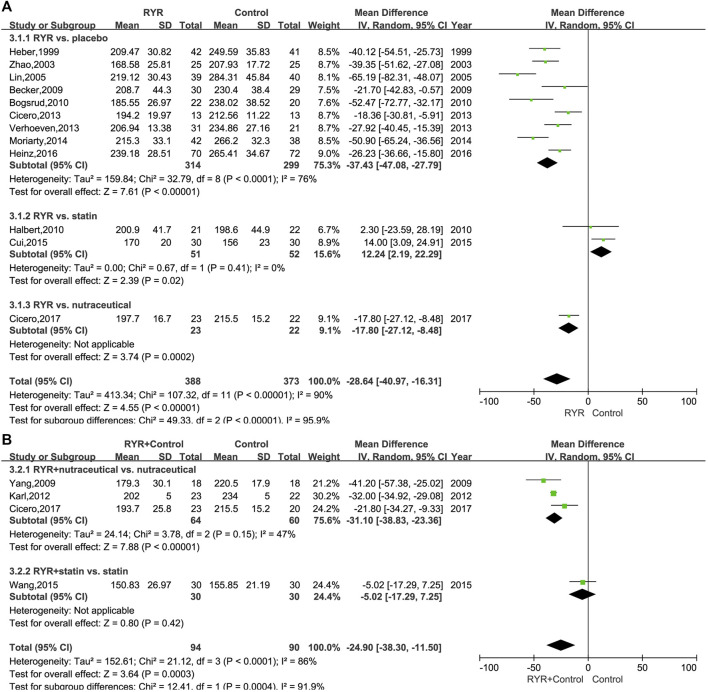
**(A)** Forest plot of TC between RYR alone and control groups. **(B)** Forest plot of TC between RYR in combination and control groups. (RYR: red yeast rice; TC: total cholesterol).

The TC levels in patients with hyperlipidemia, both when treated with RYR alone (MD, -28.64; 95% CI, −40.97 to −16.31; *p* < 0.00001; I^2^ = 90%; random effects model) and in combination (MD, −24.90; 95% CI, −38.30 to −11.50; *p* = 0.0003; I^2^ = 86%; random effects model) were significantly lower than for patients in the control group. Additionally, compared with nutraceuticals and placebo, the RYR group showed notably reduced TC levels when RYR was used alone (Nutraceutical: MD, −17.80; 95% CI, −27.12 to −8.48; *p* = 0.0002. Placebo: MD, −37.43; 95% CI, −47.08 to −27.79; *p* < 0.00001). However, when compared to statins, RYR used alone decreased the TC levels significantly less (MD, 12.24; 95% CI, 2.19 to 22.29; *p* = 0.02) (Figure 3A). Interestingly, when combining RYR with statins in the treatment of hyperlipidemia, there was no significant difference compared with using statins alone (MD, -5.02; 95% CI, −17.29 to 7.25; *p* = 0.42), while when combining RYR with a nutraceutical significantly reduced the level of TC compared with nutraceuticals alone (MD, −31.10; 95% CI, −38.83 to −23.36; *p* < 0.00001) (Figure 3B).

### Triglyceride

**FIGURE 4:**
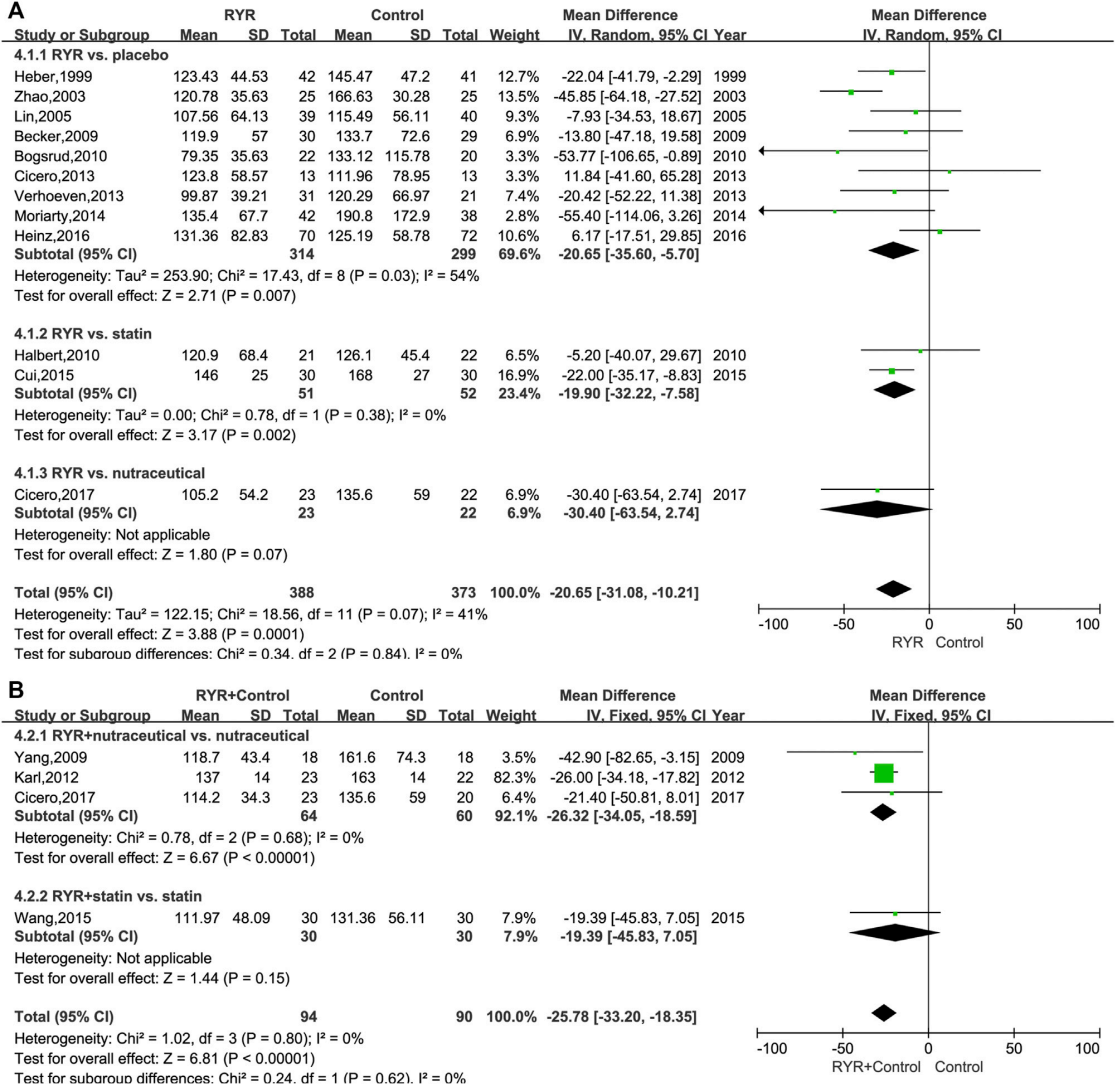
**(A)** Forest plot of TG between RYR alone and control groups. **(B)** Forest plot of TG between RYR in combination and control groups. (RYR: red yeast rice; TG: triglyceride).

The TG levels in patients with hyperlipidemia who were treated with either RYR alone (MD, −20.65; 95% CI, −31.08 to −10.21; *p* = 0.0001; I^2^ = 41%; random effects model) or in combination (MD, −25.78; 95% CI, −33.20 to −18.35; *p* < 0.00001; I^2^ = 0%; fixed effects model) were significantly lower than for patients in the control group. Additionally, for statin and placebo, the RYR group had remarkably reduced TG levels when RYR alone was used (Statin: MD, −19.90; 95% CI, −32.22 to −7.58; *p* = 0.002. Placebo: MD, −20.65; 95% CI, −35.60 to −5.70; *p* = 0.007). However, RYR showed similar results to the nutraceutical regarding a decrease in TG levels (MD, −30.40; 95% CI, −63.54 to 2.74; *p* = 0.07) (Figure 4A). When combining RYR with statins in the treatment of hyperlipidemia, there was no significant difference compared with statins alone (MD, −19.39; 95% CI, −45.83 to 7.05; *p* = 0.15). However, patients treated with RYR in combination with a nutraceutical showed significantly reduced levels of TG compared with the nutraceutical alone (MD, −26.32; 95% CI, −34.05 to −18.59; *p* < 0.00001) (Figure 4B).

### High Density Lipoprotein Cholesterol

When RYR alone was used to treat hyperlipidemia, it significantly increased HDL-C levels in patients compared with using nutraceuticals and placebo (Nutraceutical: MD, 7.60; 95% CI, 4.33 to 10.87; *p* < 0.00001. Placebo: MD, 3.47; 95% CI, 0.94 to 6.00; *p* = 0.007; I^2^ = 46%; random effects model), and was comparable to using a statin (MD, 2.50; 95% CI, −4.21 to 9.22; *p* = 0.46; I^2^ = 41%; random effects model) (Supplementary Figure S2). On the other hand, when combining RYR with statins in the treatment of hyperlipidemia, there was no significant difference compared with statins alone (MD, 3.47; 95% CI, −2.07 to 9.01; *p* = 0.22). However, patients treated with RYR in combination with a nutraceutical showed significantly increased levels of HDL-C compared with the nutraceutical alone (MD, 0.92; 95% CI, 0.13 to 1.71; *p* = 0.02; I^2^ = 20%; fixed effects model) (Supplementary Figure S3).

### Apolipoprotein

Only five trials (Zhao et al., 2003; Lin et al., 2005; Bogsrud et al., 2010; Cicero et al., 2013; Moriarty et al., 2014) included apoA-I and apoB in their measurements. Our meta-analysis of these trials indicated that RYR reduced the level of apoB significantly, but did not remarkably affect apoA-I levels [apoA-I: MD, 7.66; 95% CI, -1.11 to 16.42; *p* = 0.09; I^2^ = 67%; random effects model (Supplementary Figure S4). apoB: MD, -27.98; 95% CI, -35.51 to -20.45; *p* < 0.00001; I^2^ = 59%; random effects model (Supplementary Figure S5)].

### Safety

**FIGURE 5:**
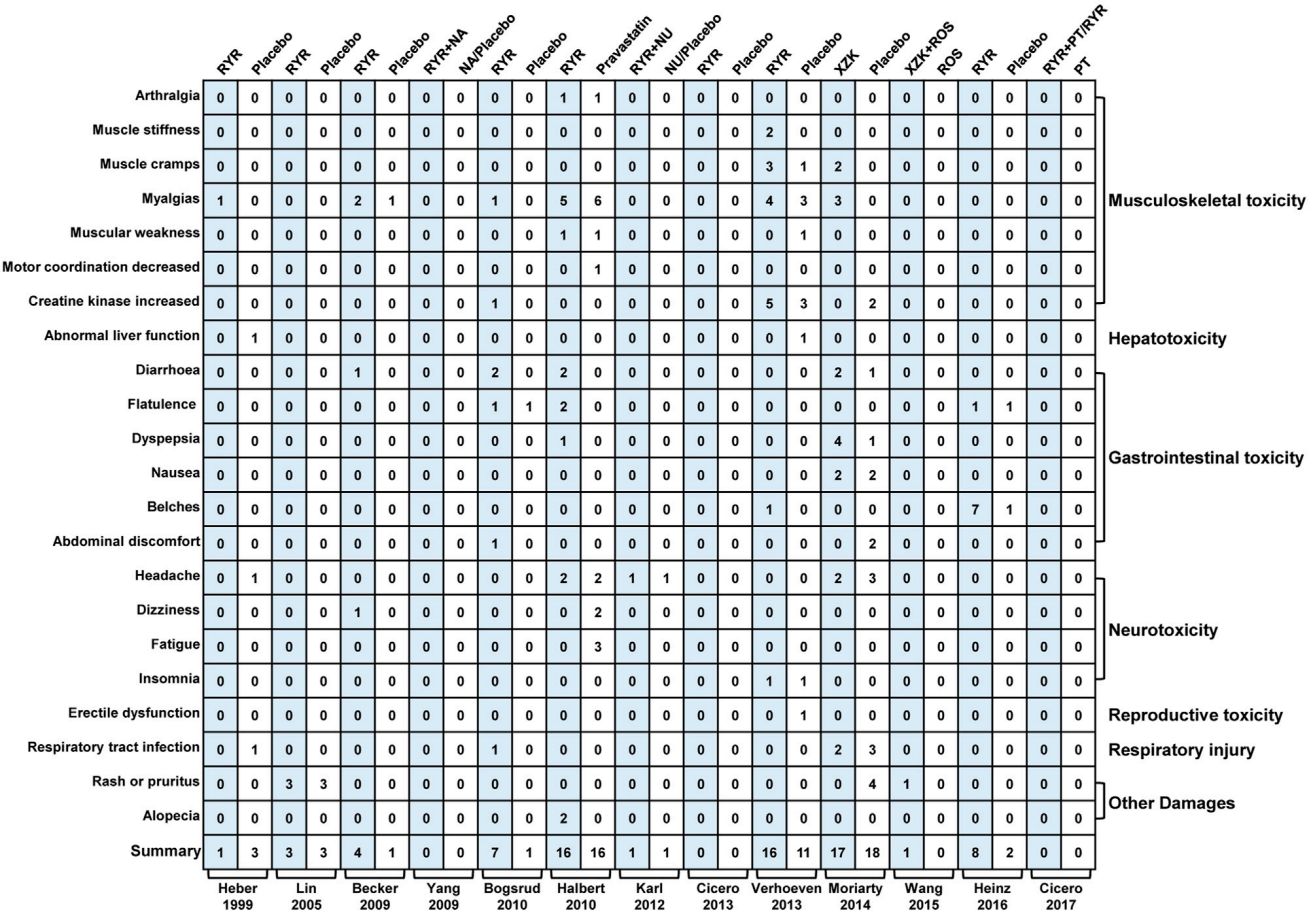
Mapping of adverse events between RYR and placebo groups. (RYR: red yeast rice; NA: nattokinase; NU: nutraceutical; XZK: xue-zhi-kang capsule; PT: phytosterols; ROS: rosuvastatin).

Thirteen trials (Heber et al., 1999; Lin et al., 2005; Becker et al., 2009; Yang et al., 2009; Bogsrud et al., 2010; Halbert et al., 2010; Karl et al., 2012; Cicero et al., 2013, Verhoeven et al., 2013; Moriarty et al., 2014; Wang et al., 2015; Heinz et al., 2016; Cicero et al., 2017) assessed adverse events (AEs) including musculoskeletal toxicity, hepatotoxicity, gastrointestinal toxicity, neurotoxicity, reproductive toxicity, respiratory injury, rash, pruritus, and alopecia. Meta-analysis results showed that the incidence of AEs in patients treated with RYR was similar to that in the control groups (RYR alone: RR, 1.18; 95% CI, 0.91 to 1.54; *p* = 0.21; I^2^ = 33%; fixed effects model. RYR combination: RR, 1.63; 95% CI, 0.22 to 11.83; *p* = 0.63; I^2^ = 0%; fixed effects model). More details regarding safety are shown in Figure 5.

## Discussion

In this systematic review we used 15 randomized controlled trials to evaluate the efficacy and safety of RYR in the treatment of hyperlipidemia. RYR at 200–4800 mg daily was effective and safe for patients with hyperlipidemia. The comprehensive results showed that, when compared with statins, RYR was more effective in lowering TG, less effective in lowering TC, and comparable with regard to lowering LDL-C and elevating HDL-C levels. Compared with nutraceuticals, RYR significantly reduced TC, LDL-C, and elevated HDL-C. Moreover, when used in combination, RYR showed a synergistic effect with nutraceutical, more effectively reducing TC, LDL-C, and TG. In addition, RYR significantly reduced apoB, but not ApoA-I.

RYR is an ancient and traditional compound used both as medicine and as food, which was first recorded in the book of the *Local Chronicles of Gutian*, dating back to the Tang Dynasty (A.D. 618–907) (Lin, 2017). Several chemical components are present in RYR, including monacolins, pigments, amino acids, organic acids, sterols, organic acids, decalin derivatives, lignans, polysaccharides, coumarin, flavonoids, and terpenoids. A more detailed list of the components and their chemical structures can be found in Supplementary Table S3.

The hypolipidemic effect of RYR can be mainly attributed to the enrichment of monacolins and pigments (Zhou et al., 2019). A randomized, double-blind, placebo-controlled clinical trial showed that using monacolins (10 mg) for a short period (4 weeks) can reduce TC, LDL-C, and HDL-C levels markedly, and suggested that the mechanism may be via an increase in mRNA expression of lipoprotein lipase and LDL-receptor (Chen, 2004). Moreover, the pigments (yellow, orange, and red) in RYR act on primary receptors involved in metabolism of cholesterol and homeostasis of bile acid (farnesoid-X receptor and peroxisome-proliferator-activated receptor-γ), upregulating their mRNA levels, in turn suppressing hepatic lipid accumulation and steatosis, promoting fecal cholesterol, triacylglycerol, and bile acid excretion. This ultimately leads to an improvement in lipid levels and alleviation of lipid metabolism disorders (Zhou et al., 2019). Our results showed that RYR can reduce levels of triglyceride-rich lipoproteins, which are the precursors of atherogenic LDL-C with smaller size and higher density. This is of great clinical relevance, since small dense LDL-C are the most atherogenic LDL-C particles and strongly associated with cardiovascular risk, due to their reduced affinity to LDL-C receptor, greater arterial entry and retention as well as enhanced susceptibility to oxidation (Rizzo and Berneis, 2007).

A large number of pharmacokinetic studies have been conducted on monacolins, as early as the 20th century (Serajuddin et al., 1991). It is known that monacolins are metabolized primarily in the gut and liver by transmembrane efflux via the drug transporter P-glycoprotein, and that they exhibit poor oral bioavailability (<5%) due to their low water solubility (1.3 μg/ml) (Chen et al., 2005; Wu et al., 2011). This means that increasing the dissolution and/or decreasing the pre-systemic clearance of these molecules is an effective approach to increasing their overall bioavailability (Chen et al., 2005). In addition, when compared to monacolins alone, the RYR compound extract is more effective in inhibiting the activity of CYP450 enzymes and P-glycoprotein, and shows a higher absorption and dissolution rate (Chen et al., 2012, 2013). Chen and others have demonstrated that participants treated with RYR had greater area under the plasma concentration time curve and maximum plasma concentration values, indicating that the oral bioavailability of monacolins in this form was significantly improved due to the increased solubility (Chen et al., 2013). Moreover, Leone and others (Leone et al., 2016) prepared a new RYR formulation including a combination of 60% gelatin with 40% alginate. Using this, they observed a delayed release of monacolins from RYR, long-term inhibition of 3-hydroxy-3-methylglutaryl-coenzyme A [the key enzyme in the synthesis of cholesterol (Ma et al., 2000)] reductase, and reduced cholesterol synthesis. As for pigment, Zhou and coworkers (Zhou et al., 2019) have compared the lipid-lowering activity of different pigments (yellow, orange and red) in rats, demonstrating that different pigments regulate lipid and cholesterol metabolism through different pathways. However, it is important to note that differences exist in physiology, metabolism, and gut flora between rats and humans. Until now, there is still a lack of high-quality studies regarding the pigment related lipid-lowering pathways of RYR in human trials. Therefore, further studies are required to explore the potential mechanism of action, and pharmacokinetics, of the various bioactive compounds present in RYR.

Our study shows that the safety and tolerance of RYR is similar to that of statins, which is consistent with the conclusion of previous systematic reviews (Ong and Aziz, 2016; Fogacci et al., 2019; Zhu et al., 2019). The present meta-analysis confirms recent postmarketing nutrivigilance data that liver damage associated with RYR intake is exceptional and hardly associated to RYR per se (Banach et al., 2021). Moreover, another systematic review of the safety of RYR pointed out that patients with an increased risk of adverse reactions to statins are sometimes able to tolerate RYR as an alternative (Gerards et al., 2015). Despite the statin-like mechanism of action, the risk related to low doses of RYR (containing 3–10 mg monacolin K) taken per day is minimal (Cicero et al., 2019). Moreover, Consuming RYR on a daily basis reduces LDL-C plasma levels between 15 and 25% within 6–8 weeks (Cicero et al., 2021). It is well known that cholesterol is essential for promoting cell signal transmission, maintaining the integrity of cell membrane, synthetic steroid hormones, coenzyme Q10 and vitamin D (Preiss and Sattar, 2011). The results of our study show that RYR was less effective than statins in lowering TC, which may be one reason why patients who are not able to tolerate statins can sometimes tolerate RYR. Nevertheless, there is still a lack of research on adverse reactions to RYR and their mechanisms.

We acknowledge that this systematic review has some limitations. Firstly, since we chose to include only high-quality RCTs as a starting point, the number of trials with sufficient quality selected for the study was limited. Secondly, only one trial (Cicero et al., 2017) had been registered in the Clinical Trials Registry Platform (http://www.clinicaltrials.gov/), making it impossible to use the protocol to confirm the absence of selective reporting. Thirdly, the methodological quality of the included trials was generally high (based on the Jadad scale), however, methodological defects still existed. Eight trials (Heber et al., 1999; Zhao et al., 2003; Lin et al., 2005; Yang et al., 2009; Bogsrud et al., 2010; Cui et al., 2015; Wang et al., 2015; Heinz et al., 2016) failed to report the specific randomized method, nine trials (Heber et al., 1999; Zhao et al., 2003; Lin et al., 2005; Yang et al., 2009; Bogsrud et al., 2010; Verhoeven et al., 2013; Cui et al., 2015; Wang et al., 2015; Heinz et al., 2016) failed to report the concealment method in detail, and one trial (Zhao et al., 2003) failed to report withdrawals and dropouts. In addition, part of the results of our meta-analysis had high heterogeneity, which might be caused by the fact that the RYR used in different studies was from different manufacturers, leading to the different contents of its active ingredients. Therefore, the results of these studies should be interpreted with caution. Finally, the longest study period in the studies selected for our review was only 24 weeks, and we suggest that more high-quality clinical studies with extended observation periods are needed in order to clarify the efficacy and safety of RYR as a long-term medication.

## Conclusion

In summary, RYR at 200–4800 mg daily appears to be effective and safe in the treatment of patients with hyperlipidemia. RYR is effective in reducing TC, TG, LDL-C, apoB and increasing HDL-C in patients with hyperlipidemia, and has an especially large positive impact on TG. In the future, high-quality clinical trials with longer observation periods are required to evaluate the efficacy and safety of RYR as a long-term medication.

## Data Availability

The original contributions presented in the study are included in the article/Supplementary Material, further inquiries can be directed to the corresponding authors.

## Abbreviations

RYR, red yeast rice; XZK, xue-zhi-kang capsule; RCTs, randomized control trials; PRISMA, preferred reporting items for systematic review and meta-analyses; MeSH, medical subject headings; TC, total cholesterol; TG, triglycerides; LDL-C, low-density lipoprotein cholesterol; HDL-C, high-density lipoprotein cholesterol; apoA-I, apolipoprotein A-I; apoB, apolipoprotein B; CVD, cardiovascular and cerebrovascular diseases; CI, confidence intervals; MD, mean difference; AEs, adverse events.
