# Supplementary material for: Red Yeast Rice for Hyperlipidemia: A Meta-Analysis of 15 High-Quality Randomized Controlled Trials
Source: Front Pharmacol. 2022 Jan 17;12:819482. doi: 10.3389/fphar.2021.819482 (PMC8802088; doi:10.3389/fphar.2021.819482)
Supplement: Supplementary file 1 [file DataSheet1.docx]

# Supplementary Information

**Red yeast rice for hyperlipemia: A meta-analysis of 15 high-quality randomized controlled trials**

**Supplementary Table S1: Preferred Reporting Items for Systematic Reviews and Meta-Analyses (PRISMA) checklist**

| **Section and Topic** | **Item #** | **Checklist item** | **Location where item is reported** |
| --- | --- | --- | --- |
| **TITLE** | | |  |
| Title | 1 | Identify the report as a systematic review. | **Page 1 (Title)** |
| **ABSTRACT** | | |  |
| Abstract | 2 | See the PRISMA 2020 for Abstracts checklist. | **Pages 3-4 (Abstract)** |
| **INTRODUCTION** | | |  |
| Rationale | 3 | Describe the rationale for the review in the context of existing knowledge. | **Pages 6-7 (Introduction)** |
| Objectives | 4 | Provide an explicit statement of the objective(s) or question(s) the review addresses. | **Pages 6-7 (Introduction)** |
| **METHODS** | | |  |
| Eligibility criteria | 5 | Specify the inclusion and exclusion criteria for the review and how studies were grouped for the syntheses. | **Page 8 (Search strategy)** |
| Information sources | 6 | Specify all databases, registers, websites, organisations, reference lists and other sources searched or consulted to identify studies. Specify the date when each source was last searched or consulted. | **Page 8 (Search strategy)** |
| Search strategy | 7 | Present the full search strategies for all databases, registers and websites, including any filters and limits used. | **Page 8 (Search strategy)** |
| Selection process | 8 | Specify the methods used to decide whether a study met the inclusion criteria of the review, including how many reviewers screened each record and each report retrieved, whether they worked independently, and if applicable, details of automation tools used in the process. | **Page 8-9 (Inclusion and exclusion criteria)** |
| Data collection process | 9 | Specify the methods used to collect data from reports, including how many reviewers collected data from each report, whether they worked independently, any processes for obtaining or confirming data from study investigators, and if applicable, details of automation tools used in the process. | **Page 9 (Data extraction)** |
| Data items | 10a | List and define all outcomes for which data were sought. Specify whether all results that were compatible with each outcome domain in each study were sought (e.g. for all measures, time points, analyses), and if not, the methods used to decide which results to collect. | **Page 9 (Data extraction)** |
|  | 10b | List and define all other variables for which data were sought (e.g. participant and intervention characteristics, funding sources). Describe any assumptions made about any missing or unclear information. | **Page 9 (Data extraction)** |
| Study risk of bias assessment | 11 | Specify the methods used to assess risk of bias in the included studies, including details of the tool(s) used, how many reviewers assessed each study and whether they worked independently, and if applicable, details of automation tools used in the process. | **Page 9-10 (Risk of bias in individual studies)** |
| Effect measures | 12 | Specify for each outcome the effect measure(s) (e.g. risk ratio, mean difference) used in the synthesis or presentation of results. | **Page 10 (Statistical analysis)** |
| Synthesis methods | 13a | Describe the processes used to decide which studies were eligible for each synthesis (e.g. tabulating the study intervention characteristics and comparing against the planned groups for each synthesis (item #5)). | **Page 10 (Statistical analysis)** |
|  | 13b | Describe any methods required to prepare the data for presentation or synthesis, such as handling of missing summary statistics, or data conversions. | **Page 10 (Statistical analysis)** |
|  | 13c | Describe any methods used to tabulate or visually display results of individual studies and syntheses. | **Page 10 (Statistical analysis)** |
|  | 13d | Describe any methods used to synthesize results and provide a rationale for the choice(s). If meta-analysis was performed, describe the model(s), method(s) to identify the presence and extent of statistical heterogeneity, and software package(s) used. | **Page 10 (Statistical analysis)** |
|  | 13e | Describe any methods used to explore possible causes of heterogeneity among study results (e.g. subgroup analysis, meta-regression). | **Page 10 (Statistical analysis)** |
|  | 13f | Describe any sensitivity analyses conducted to assess robustness of the synthesized results. | **Page 10 (Statistical analysis)** |
| Reporting bias assessment | 14 | Describe any methods used to assess risk of bias due to missing results in a synthesis (arising from reporting biases). | **Page 9-10 (Risk of bias assessments)** |
| Certainty assessment | 15 | Describe any methods used to assess certainty (or confidence) in the body of evidence for an outcome. | **Page 10 (Statistical analysis)** |
| **RESULTS** | | |  |
| Study selection | 16a | Describe the results of the search and selection process, from the number of records identified in the search to the number of studies included in the review, ideally using a flow diagram. | **Pages 10-11 (Study selection of included studies)** |
|  | 16b | Cite studies that might appear to meet the inclusion criteria, but which were excluded, and explain why they were excluded. | **Pages 10-11 (Study selection of included studies)** |
| Study characteristics | 17 | Cite each included study and present its characteristics. | **Pages 11-12 (study characteristics)** |
| Risk of bias in studies | 18 | Present assessments of risk of bias for each included study. | **Pages 12-13 (Risk of bias assessments)** |
| Results of individual studies | 19 | For all outcomes, present, for each study: (a) summary statistics for each group (where appropriate) and (b) an effect estimate and its precision (e.g. confidence/credible interval), ideally using structured tables or plots. | **Pages 13-16 (Primary outcomes / Secondary outcomes)** |
| Results of syntheses | 20a | For each synthesis, briefly summarise the characteristics and risk of bias among contributing studies. | **Pages 13-16 (Primary outcomes / Secondary outcomes)** |
|  | 20b | Present results of all statistical syntheses conducted. If meta-analysis was done, present for each the summary estimate and its precision (e.g. confidence/credible interval) and measures of statistical heterogeneity. If comparing groups, describe the direction of the effect. | **Pages 13-16 (Primary outcomes / Secondary outcomes)** |
|  | 20c | Present results of all investigations of possible causes of heterogeneity among study results. | **Pages 13-16 (Primary outcomes / Secondary outcomes)** |
|  | 20d | Present results of all sensitivity analyses conducted to assess the robustness of the synthesized results. | **Pages 13-16 (Primary outcomes / Secondary outcomes)** |
| Reporting biases | 21 | Present assessments of risk of bias due to missing results (arising from reporting biases) for each synthesis assessed. | **Pages 12-13 (Risk of bias assessments)** |
| Certainty of evidence | 22 | Present assessments of certainty (or confidence) in the body of evidence for each outcome assessed. | **Pages 13-16 (Primary outcomes / Secondary outcomes)** |
| **DISCUSSION** | | |  |
| Discussion | 23a | Provide a general interpretation of the results in the context of other evidence. | **Pages 16-20 (Discussion)** |
|  | 23b | Discuss any limitations of the evidence included in the review. | **Pages 19-20 (Limitations)** |
|  | 23c | Discuss any limitations of the review processes used. | **Pages 19-20 (Limitations)** |
|  | 23d | Discuss implications of the results for practice, policy, and future research. | **Pages 16-20 (Discussion)** |
| **OTHER INFORMATION** | | |  |
| Registration and protocol | 24a | Provide registration information for the review, including register name and registration number, or state that the review was not registered. | **Page 8 (Methods)** |
|  | 24b | Indicate where the review protocol can be accessed, or state that a protocol was not prepared. | **Page 8 (Methods)** |
|  | 24c | Describe and explain any amendments to information provided at registration or in the protocol. | **Page 8 (Methods)** |
| Support | 25 | Describe sources of financial or non-financial support for the review, and the role of the funders or sponsors in the review. | **Page 22 (Funding)** |
| Competing interests | 26 | Declare any competing interests of review authors. | **Page 22 (Declaration of interest)** |
| Availability of data, code and other materials | 27 | Report which of the following are publicly available and where they can be found: template data collection forms; data extracted from included studies; data used for all analyses; analytic code; any other materials used in the review. | **Page 22 (Data Availability Statement)** |

*From:*  Page MJ, McKenzie JE, Bossuyt PM, Boutron I, Hoffmann TC, Mulrow CD, et al. The PRISMA 2020 statement: an updated guideline for reporting systematic reviews. BMJ 2021;372:n71. doi: 10.1136/bmj.n71

PRISMA: Preferred Reporting Items for Systematic Reviews and Meta-Analyse

**Supplementary Table S2: Search Strategy**

| Pubmed | 1 | red yeast rice [Supplementary Concept] |
| --- | --- | --- |
|  | 2 | red yeast rice[Title/Abstract] |
|  | 3 | red rice yeast[Title/Abstract] |
|  | 4 | cholestin[Title/Abstract] |
|  | 5 | Chinese red-yeast-rice dietary supplement[Title/Abstract] |
|  | 6 | red kojic rice[Title/Abstract] |
|  | 7 | red rice[Title/Abstract] |
|  | 8 | monascus color[Title/Abstract] |
|  | 9 | momascusc colours[Title/Abstract] |
|  | 10 | monascus red[Title/Abstract] |
|  | 11 | red rice startermomascusc colours[Title/Abstract] |
|  | 12 | ryr[Title/Abstract] |
|  | 13 | r-y-r[Title/Abstract] |
|  | 14 | riz rouge[Title/Abstract] |
|  | 15 | monacolin k[Title/Abstract] |
|  | 16 | monascus[Title/Abstract] |
|  | 17 | monascus purpureus[Title/Abstract] |
|  | 18 | monascus purpureus went[Title/Abstract] |
|  | 19 | hong qu[Title/Abstract] |
|  | 20 | xuezhikang[Title/Abstract] |
|  | 21 | 1-20/or |
|  | 22 | hyperlipidemias[Mesh] |
|  | 23 | hyperlipemia[Title/Abstract] |
|  | 24 | hyperlipemias[Title/Abstract] |
|  | 25 | hyperlipaemia[Title/Abstract] |
|  | 26 | hyperlipidemia[Title/Abstract] |
|  | 27 | hyperlipidemias[Title/Abstract] |
|  | 28 | hyperlipidaemia[Title/Abstract] |
|  | 29 | high cholesterol[Title/Abstract] |
|  | 30 | hypercholesterolemia[Title/Abstract] |
|  | 31 | lipid profile[Title/Abstract] |
|  | 32 | lipidemia[Title/Abstract] |
|  | 33 | lipidemias[Title/Abstract] |
|  | 34 | lipemia[Title/Abstract] |
|  | 35 | lipemias[Title/Abstract] |
|  | 36 | dyslipidemia[Title/Abstract] |
|  | 37 | 22-36/or |
|  | 38 | 21 and 37 |
| Embase | 1 | 'cholestin'/exp |
|  | 2 | 'xuezhikang'/exp |
|  | 3 | 'red yeast rice':ti,ab,kw |
|  | 4 | 'red rice yeast':ti,ab,kw |
|  | 5 | 'cholestin':ti,ab,kw |
|  | 6 | 'Chinese red-yeast-rice dietary supplement':ti,ab,kw |
|  | 7 | 'red kojic rice':ti,ab,kw |
|  | 8 | 'red rice':ti,ab,kw |
|  | 9 | 'monascus color':ti,ab,kw |
|  | 10 | 'momascusc colours':ti,ab,kw |
|  | 11 | 'monascus red':ti,ab,kw |
|  | 12 | 'red rice startermomascusc colours':ti,ab,kw |
|  | 13 | 'ryr':ti,ab,kw |
|  | 14 | 'r-y-r':ti,ab,kw |
|  | 15 | 'riz rouge':ti,ab,kw |
|  | 16 | 'monacolin k':ti,ab,kw |
|  | 17 | 'monascus':ti,ab,kw |
|  | 18 | 'monascus purpureus':ti,ab,kw |
|  | 19 | 'monascus purpureus went':ti,ab,kw |
|  | 20 | 'hong qu':ti,ab,kw |
|  | 21 | 'xuezhikang':ti,ab,kw |
|  | 22 | 1-21/or |
|  | 23 | 'hyperlipidemia'/exp |
|  | 24 | 'hyperlipemia':ti,ab,kw |
|  | 25 | 'hyperlipemias':ti,ab,kw |
|  | 26 | 'hyperlipaemia':ti,ab,kw |
|  | 27 | 'hyperlipidemia':ti,ab,kw |
|  | 28 | 'hyperlipidemias':ti,ab,kw |
|  | 29 | 'hyperlipidaemia':ti,ab,kw |
|  | 30 | 'high cholesterol':ti,ab,kw |
|  | 31 | 'hypercholesterolemia':ti,ab,kw |
|  | 32 | 'lipid profile':ti,ab,kw |
|  | 33 | 'lipidemia':ti,ab,kw |
|  | 34 | 'lipidemias':ti,ab,kw |
|  | 35 | 'lipemia':ti,ab,kw |
|  | 36 | 'lipemias':ti,ab,kw |
|  | 37 | 'dyslipidemia':ti,ab,kw |
|  | 38 | 23-37/or |
|  | 39 | 22 and 38 |
| Cochrane | 1 | MeSH descriptor:[red yeast rice] explode all trees |
|  | 2 | (red yeast rice):ti,ab,kw |
|  | 3 | (red rice yeast):ti,ab,kw |
|  | 4 | (cholestin):ti,ab,kw |
|  | 5 | (Chinese red-yeast-rice dietary supplement):ti,ab,kw |
|  | 6 | (red kojic rice):ti,ab,kw |
|  | 7 | (red rice):ti,ab,kw |
|  | 8 | (monascus color):ti,ab,kw |
|  | 9 | (momascusc colours):ti,ab,kw |
|  | 10 | (monascus red):ti,ab,kw |
|  | 11 | (red rice startermomascusc colours):ti,ab,kw |
|  | 12 | (ryr):ti,ab,kw |
|  | 13 | (r-y-r):ti,ab,kw |
|  | 14 | (riz rouge):ti,ab,kw |
|  | 15 | (monacolin k):ti,ab,kw |
|  | 16 | (monascus):ti,ab,kw |
|  | 17 | (monascus purpureus):ti,ab,kw |
|  | 18 | (monascus purpureus went):ti,ab,kw |
|  | 19 | (hong qu):ti,ab,kw |
|  | 20 | (xuezhikang):ti,ab,kw |
|  | 21 | 1-20/or |
|  | 22 | MeSH descriptor:[hyperlipidemias] explode all trees |
|  | 23 | (hyperlipemia):ti,ab,kw |
|  | 24 | (hyperlipemias):ti,ab,kw |
|  | 25 | (hyperlipaemia):ti,ab,kw |
|  | 26 | (hyperlipidemia):ti,ab,kw |
|  | 27 | (hyperlipidemias):ti,ab,kw |
|  | 28 | (hyperlipidaemia):ti,ab,kw |
|  | 29 | (high cholesterol):ti,ab,kw |
|  | 30 | (hypercholesterolemia):ti,ab,kw |
|  | 31 | (lipid profile):ti,ab,kw |
|  | 32 | (lipidemia):ti,ab,kw |
|  | 33 | (lipidemias):ti,ab,kw |
|  | 34 | (lipemia):ti,ab,kw |
|  | 35 | (lipemias):ti,ab,kw |
|  | 36 | (dyslipidemia):ti,ab,kw |
|  | 37 | 22-36/or |
|  | 38 | 21 and 37 |

**Supplementary Table S3: Molecular structure of main chemical constituents of red yeast rice**

| Class | Compound | CAS registry number | Molecular Formula | Structure |
| --- | --- | --- | --- | --- |
| Pigments | Rubropunctamine | 13552-06-2 | C_21_H_23_NO_4_ | 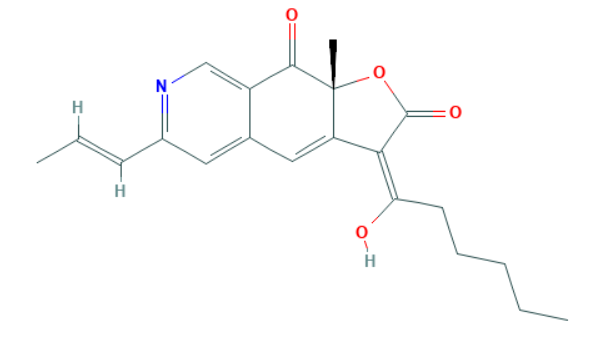 |
|  | Rubropunctatin | 13471-84-6 | C_21_H_22_O_5_ | 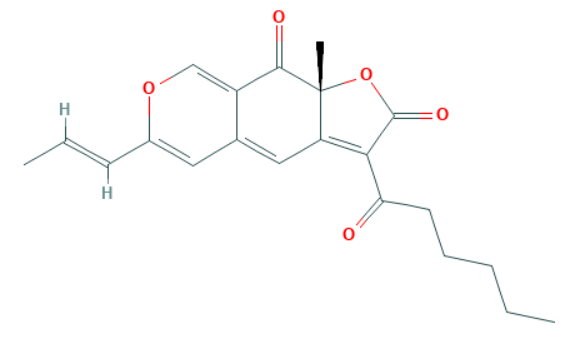 |
|  | Monascorubramine | 1003392-65-1 | C_23_H_27_NO_4_ | 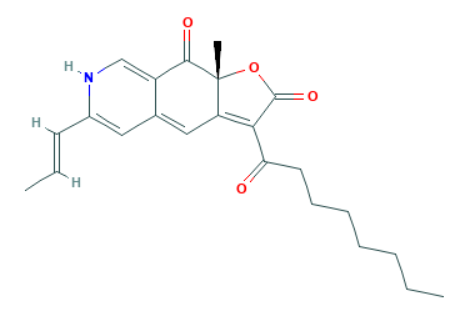 |
|  | Monascorubrin | 13283-85-7 | C_23_H_26_O_5_ | 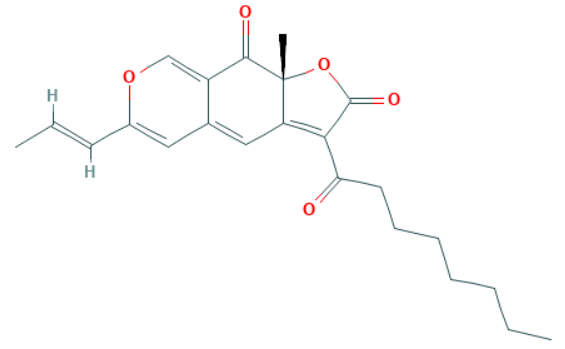 |
|  | Monascin | 3567-98-4 | C_21_H_26_O_5_ | 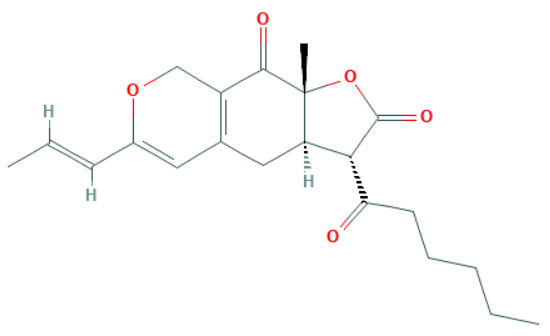 |
|  | Ankaflavin | 50980-32-0 | C_23_H_30_O_5_ | 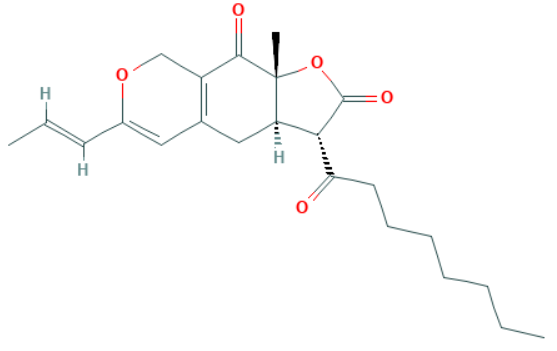 |
|  | Xanthomonasin A | 140375-37-7 | C_21_H_24_O_7_ | 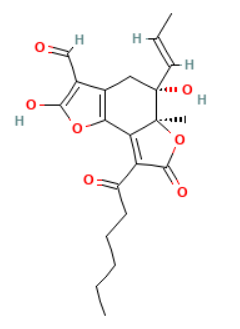 |
|  | Xanthomonasin B | 146445-98-9 | C_23_H_28_O_7_ | 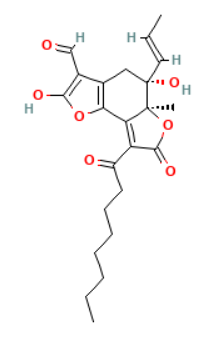 |
|  | Monankarin A | 182161-52-0 | C_20_H_22_O_6_ | 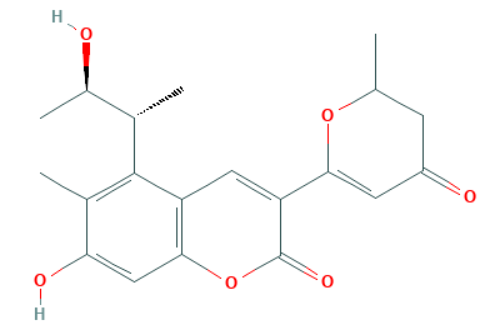 |
|  | Monasfluore A | 1004537-75-0 | C_21_H_24_O_5_ | 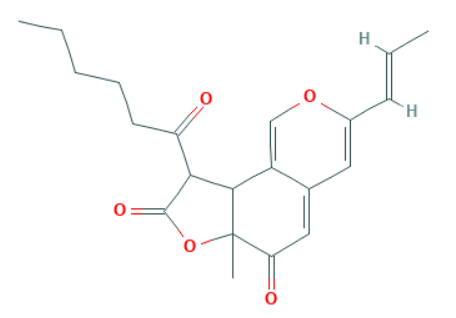 |
|  | Monasfluore B | 1004537-76-1 | C_23_H_28_O_5_ | 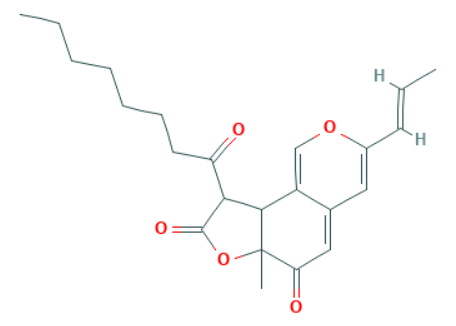 |
|  | Monapurone A | 1263777-47-4 | C_20_H_26_O_4_ | 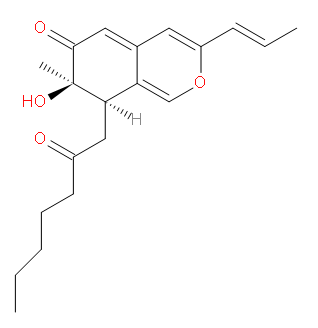 |
|  | Monapurone B | 1263777-50-9 | C_21_H_28_O_4_ | 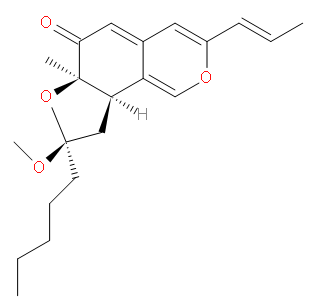 |
|  | Monapurone C | 1263777-48-5 | C_21_H_28_O_4_ | 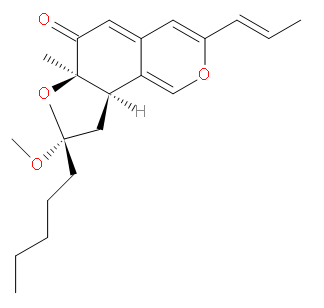 |
|  | Monascopyridine A | 604786-54-1 | C_21_H_25_NO_4_ | 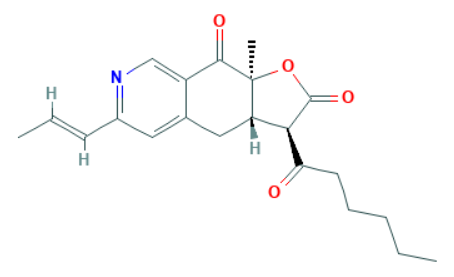 |
|  | Monascopyridine B | 604786-55-2 | C_23_H_29_NO_4_ | 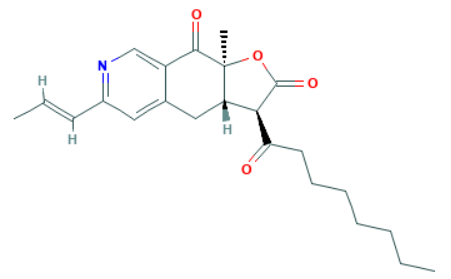 |
|  | Monascopyridine C | 909094-27-5 | C_20_H_27_NO_3_ | 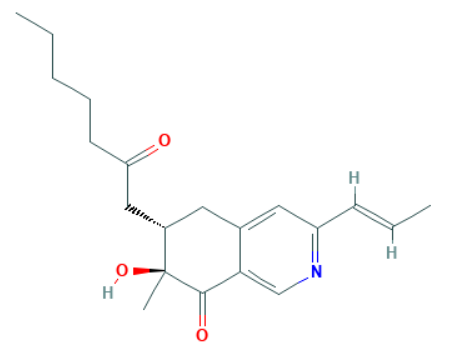 |
|  | Monascopyridine D | 909094-28-6 | C_22_H_31_NO_3_ | 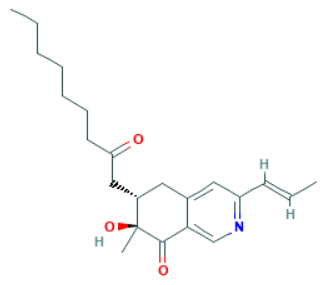 |
|  | Monascopyridine E | 1313735-11-3 | C_21_H_27_NO_4_ | 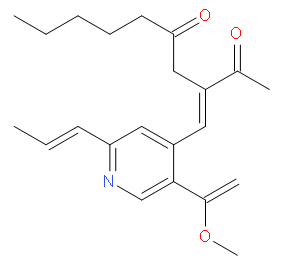 |
|  | Monascopyridine F | 1313735-13-5 | C_23_H_31_NO_4_ | 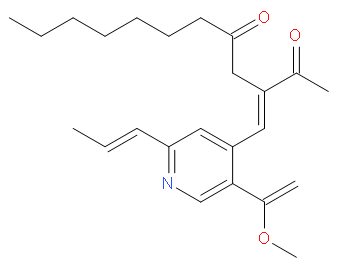 |
|  | Monapurfluore A | 1259424-24-2 | C_23_H_32_O_4_ | 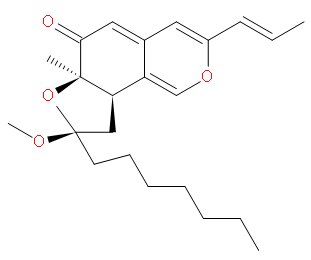 |
|  | Monapurfluore B | 1259424-22-0 | C_23_H_32_O_4_ | 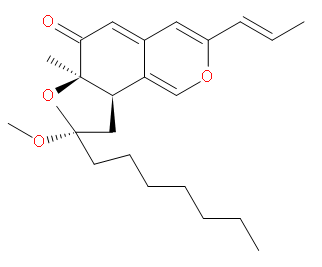 |
|  | 4-[2,4-Dihydroxy-6-(3-hydroxybutanethioyloxy)-3-methylphenyl]-3,4-dihydroxy-3,6-dimethylheptanoic acid | 910788-93-1 | C_20_H_30_O_8_S | 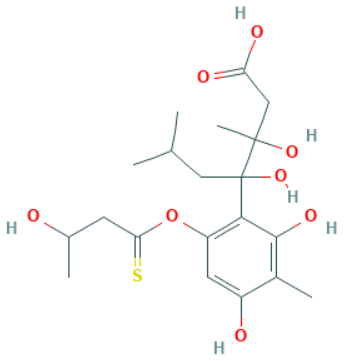 |
|  | 9-Hexanoyl-3-(2-hydroxypropyl)-6a-methyl-9,9a-dihydro-6H-furo[2,3-h]isochromene-6,8(6aH)-dione | 910788-92-0 | C_21_H_26_O_6_ | 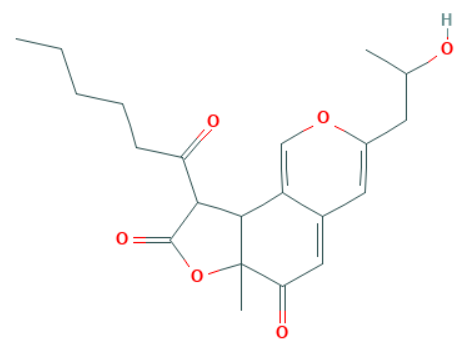 |
|  | Monapilosusazaphilone | 1444202-34-9 | C_23_H_32_O_5_ | 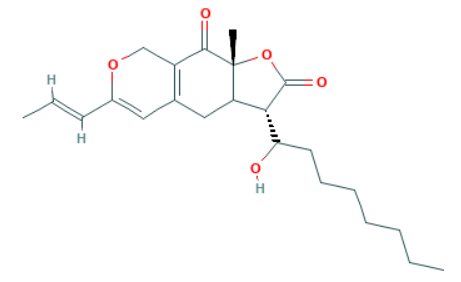 |
| Monacolins | Monacolin K | 75330-75-5 | C_24_H_36_O_5_ | 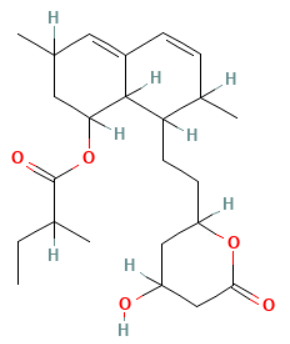 |
|  | Monacolin L | 79394-47-1 | C_19_H_28_O_3_ | 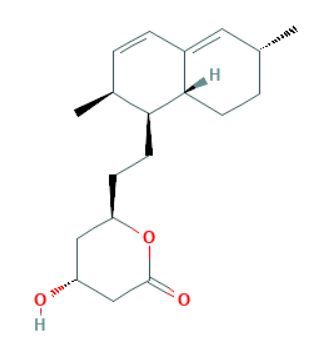 |
|  | Monacolin Q | 1879038-89-7 | C_19_H_22_O_2_ | 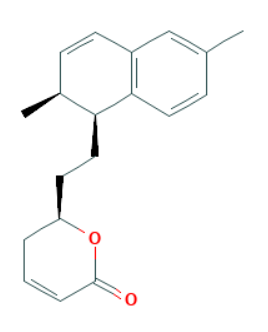 |
|  | Monacolin R | 1879038-90-0 | C_19_H_28_O_3_ | 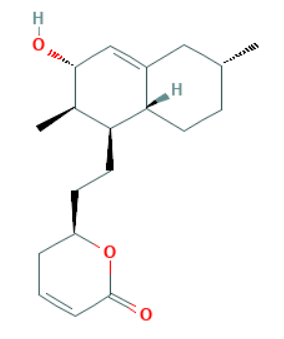 |
|  | Monacolin S | 81693-02-9 | C_24_H_38_O_7_ | 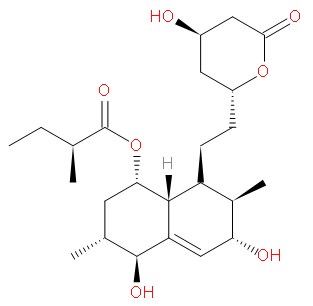 |
|  | Dehydromonacolin J | 1355394-51-2 | C_19_H_26_O_3_ | 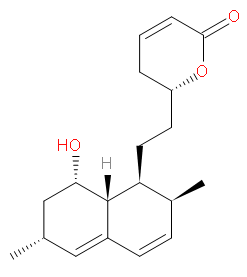 |
|  | Dehydromonacolin K | 109273-98-5 | C_24_H_34_O_4_ | 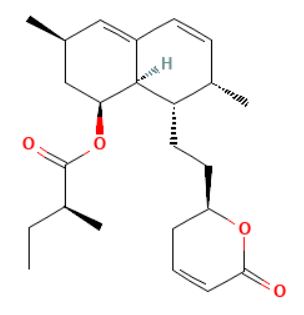 |
|  | Dehydromonacolin L | 1355394-52-3 | C_19_H_26_O_2_ | 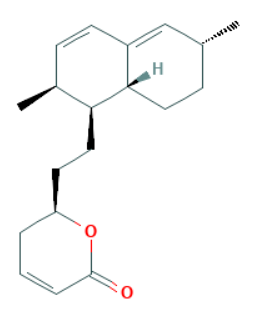 |
|  | Dehydromonacolin N | 1355394-50-1 | C_21_H_28_O_4_ | 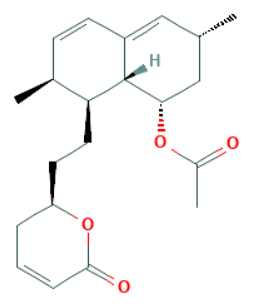 |
|  | Dihydromonacolin K | 77517-29-4 | C_24_H_38_O_5_ | 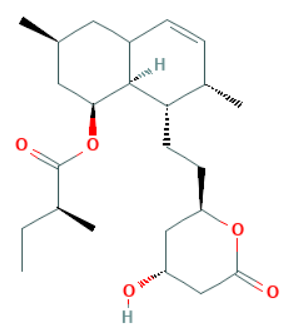 |
|  | Dihydromonacolin L | 86827-77-2 | C_19_H_30_O_3_ | 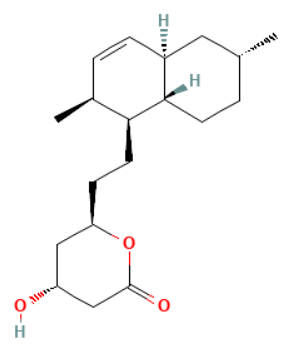 |
|  | Dihydromonacolin-MV | 935846-59-6 | C_24_H_38_O_5_ | 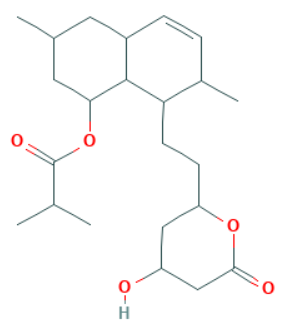 |
|  | Dehydromonacolin-MV2 | 1018346-91-2 | C_19_H_26_O_5_ | 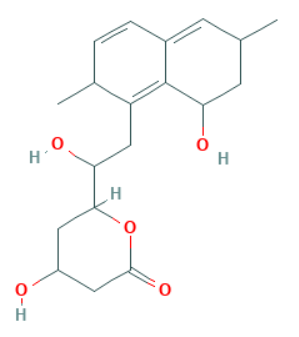 |
|  | Ethyl ester of monacolin K | 77517-31-8 | C_26_H_42_O_6_ | 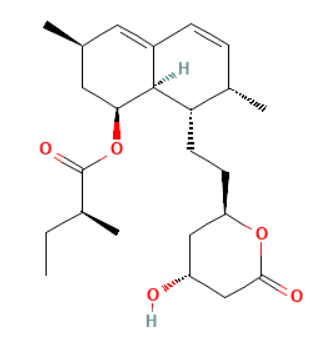 |
|  | Methyl ester of the hydroxyl acid form of monacolin K | 77934-80-6 | C_25_H_40_O_6_ | 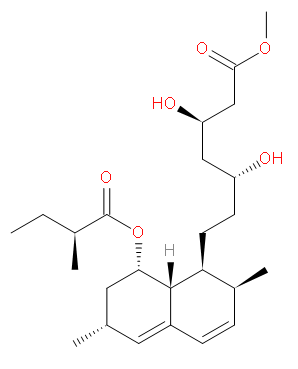 |
|  | Methyl ester of the hydroxy acid form of monacolin L | 312710-94-4 | C_20_H_32_O_4_ | 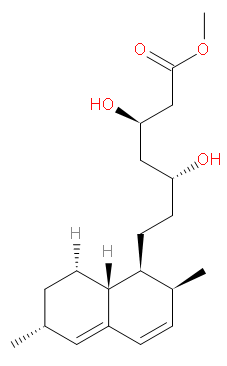 |
|  | α,β-Dehydromonacolin S | 1879038-91-1 | C_24_H_36_O_6_ | 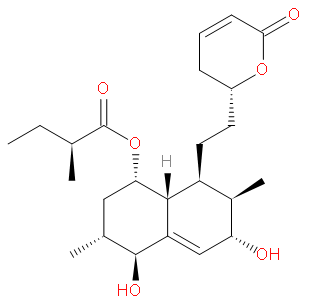 |
|  | α,β-Hydromonacolin Q | 118045-32-2 | C_19_H_24_O_3_ | 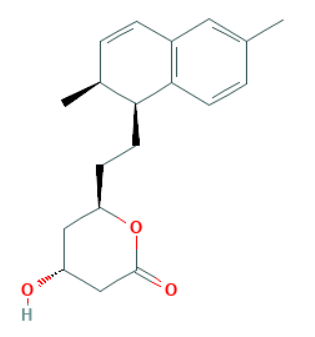 |
|  | 3α-Hydroxy-3,5-dihydromonacolin L | 119786-66-2 | C_19_H_30_O_4_ | 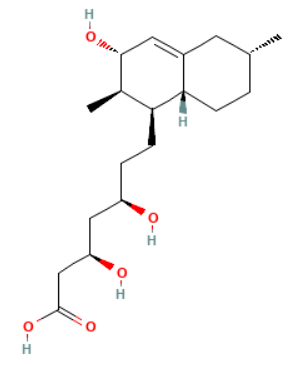 |
|  | 3β-Hydroxy-3,5-dihydromonacolin L | 1879038-92-2 | C_19_H_30_O_4_ | 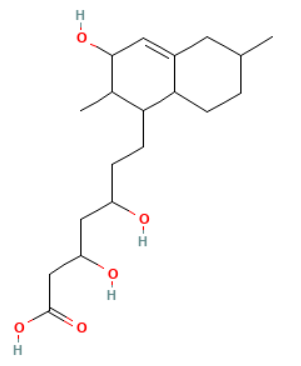 |
|  | α,β-Dehydrodihydromonacolin K | 312710-92-2 | C_24_H_36_O_4_ | 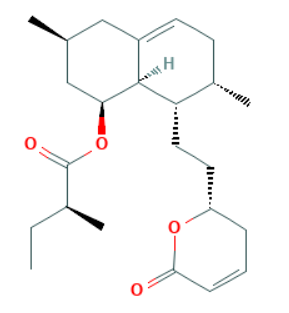 |
|  | α,β-Dehydrodihydromonacolin L | 531523-94-1 | C_19_H_28_O_2_ | 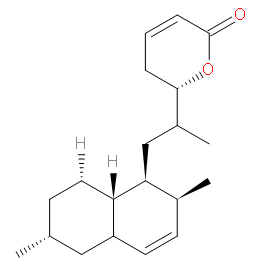 |
|  | (1S,2S,4aR,6S,8-S,8aS,3′S,5′R,2″S)-Methyl 1,2,4a,5,6,7,8,8a-octahydro-3′,5′-dihydroxy-2,6-dimethyl-8-[(2-methyl-1-oxobutyl)oxy]-1-naphthaleneheptanoate | 101834-04-2 | C_25_H_42_O_6_ | 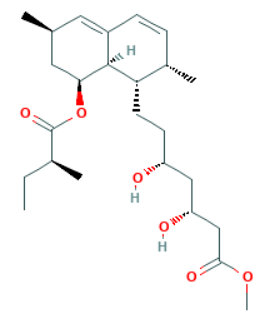 |
| Organic acids and amino acids | Linoleic acid | 60-33-3 | C_18_H_32_O_2_ | 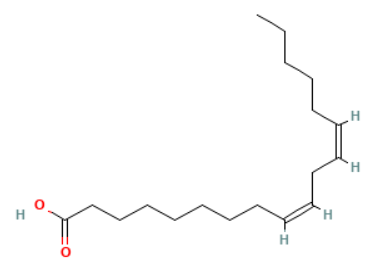 |
|  | α-Linolenic acid | 463-40-1 | C_18_H_30_O_2_ | 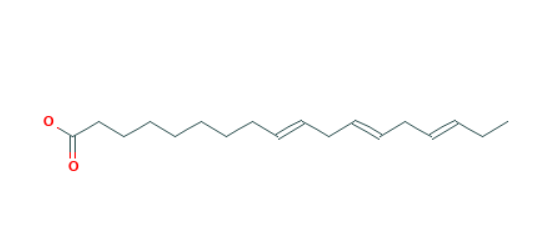 |
|  | Citrinin | 1086-03-9 | C_13_H_14_O_5_ | 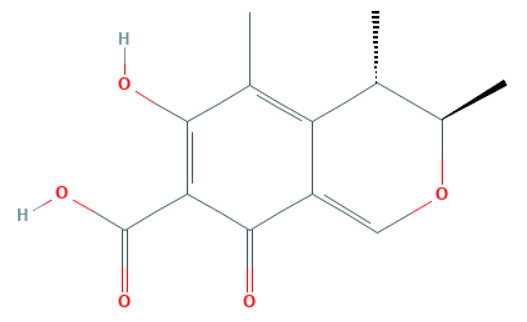 |
|  | 1-Heptadecanecarboxylic acid | 57-11-4 | C_18_H_36_O_2_ | 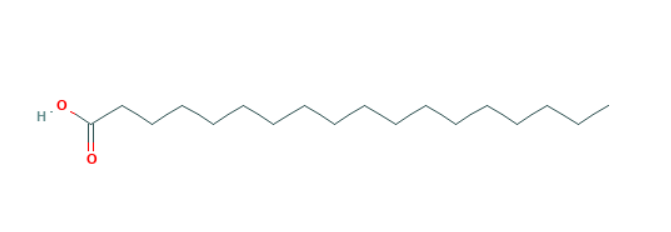 |
|  | 1-Pentadecanecarboxylic acid | 57-10-3 | C_16_H_32_O_2_ | 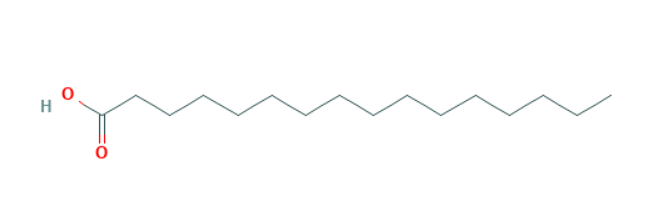 |
|  | 2-Hydroxyoctadecanoic acid | 629-22-1 | C_18_H_36_O_3_ | 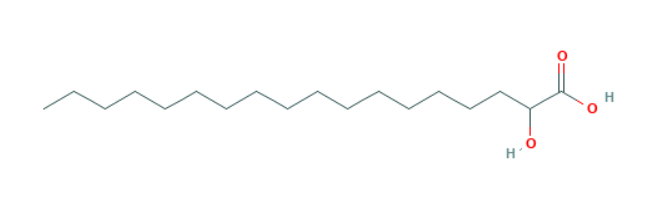 |
|  | 5-(2′-Hydroxy-6′-methyl phenyl)-3-methylfuran-2-carboxylic acid | 2060554-52-9 | C_13_H_12_O_4_ | 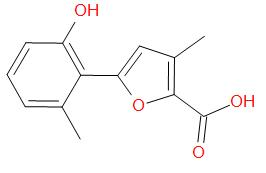 |
|  | (+)-Monascumic acid | 673477-38-8 | C_10_H_17_NO_4_ | 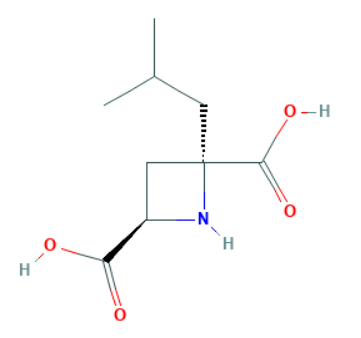 |
|  | (−)-Monascumic acid | 673477-39-9 | C_10_H_17_NO_4_ | 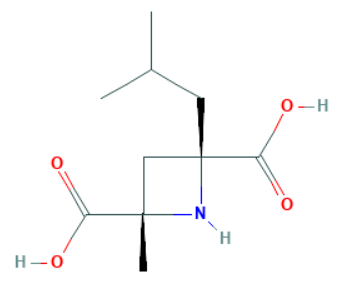 |
| Sterols | Ergosterol | 57-87-4 | C_28_H_44_O | 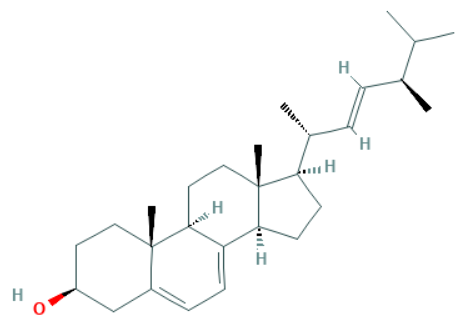 |
|  | Stigmasterol | 83-48-7 | C_29_H_48_O | 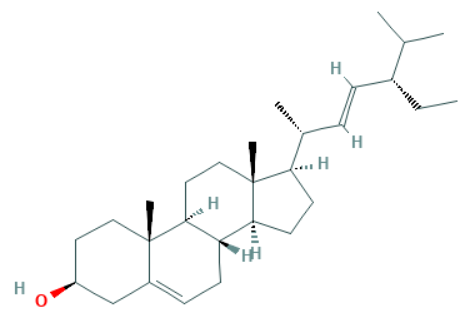 |
|  | β-Sitosterol | 83-46-5 | C_29_H_50_O | 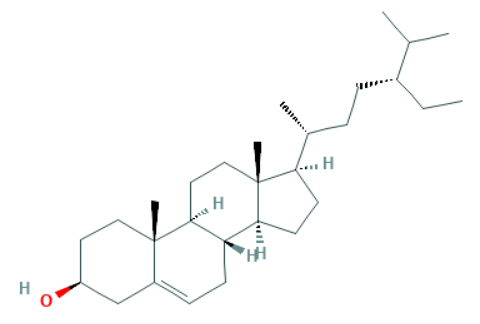 |
|  | 3β-Hydroxylstigmast-5-en-7-one | 2034-74-4 | C_29_H_48_O_2_ | 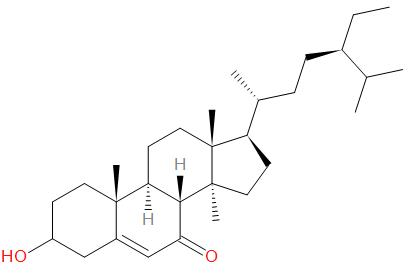 |
|  | 3β-Hydroxystigmasta-5,22-dien-7-one | 36449-99-7 | C_29_H_46_O_2_ | 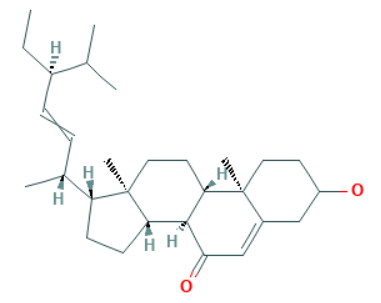 |
|  | 6β-Hydroxystigmast-4-en-3-one | 36450-02-9 | C_29_H_48_O_2_ | 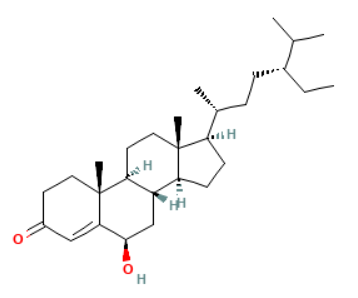 |
|  | 6β-Hydroxystigmasta-4,22-dien-3-one | 36450-01-8 | C_29_H_46_O_2_ | 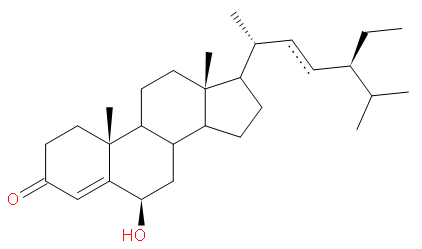 |
|  | Daucosterol | 474-58-8 | C_35_H_60_O_6_ | 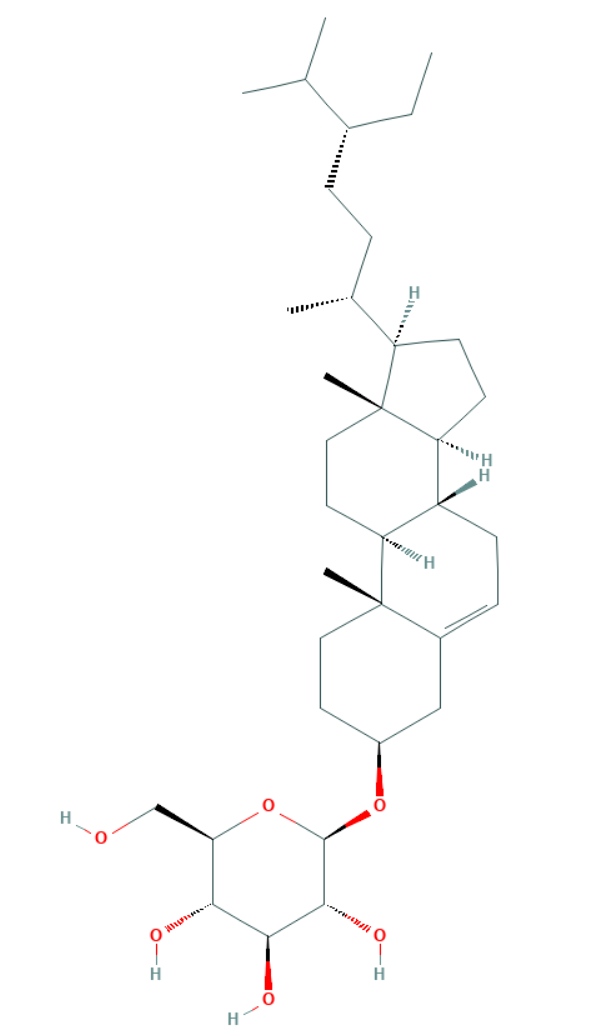 |
|  | β-Sitosteryl palmitate | 110716-42-2 | C_44_H_76_O_2_ | 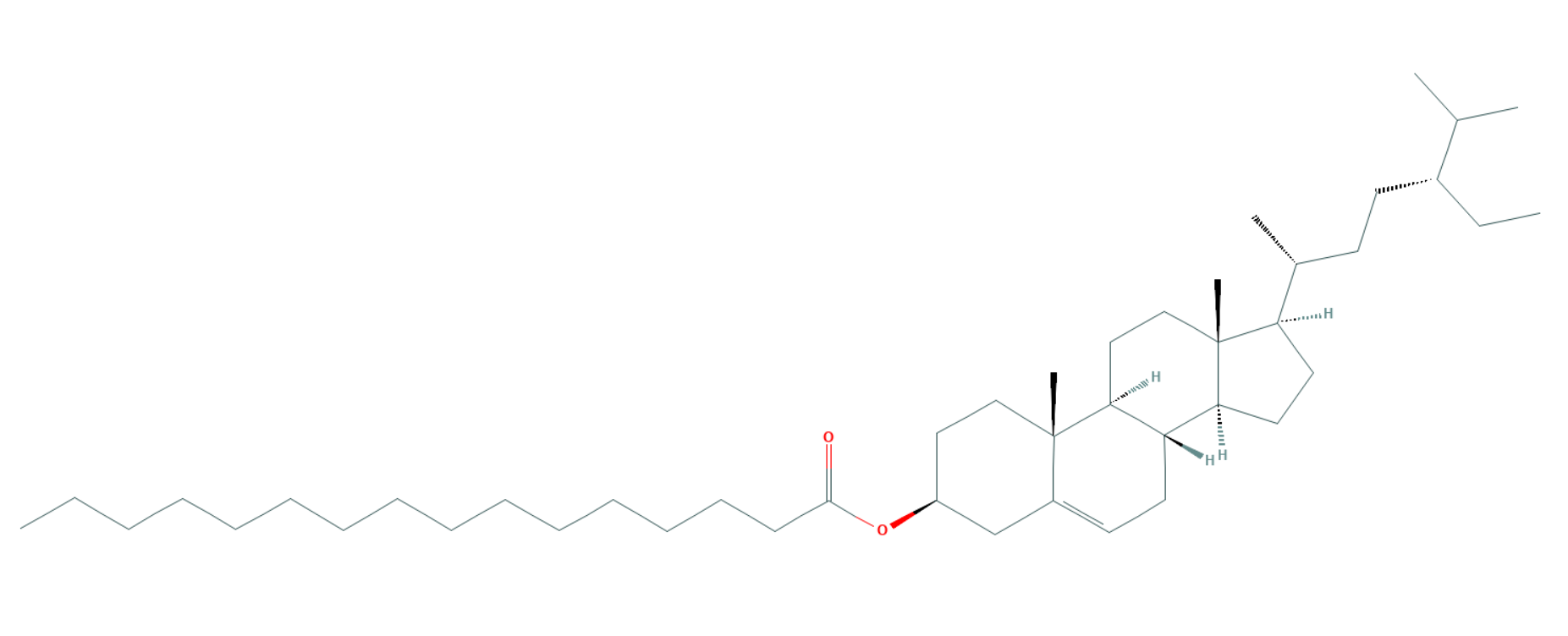 |
| Decalin derivatives | Monascusic acid A | 1364517-38-3 | C_15_H_22_O_2_ | 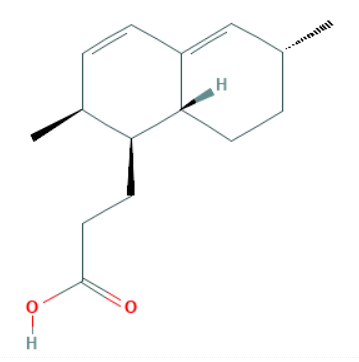 |
|  | Monascusic acid B | 1364517-30-5 | C_15_H_22_O_2_ | 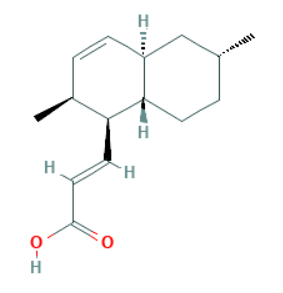 |
|  | Monascusic acid C | 1364517-32-7 | C_15_H_22_O_2_ | 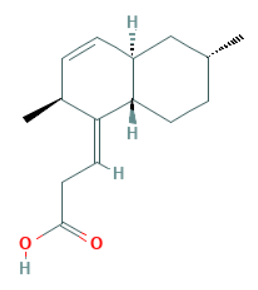 |
|  | Monascusic acid D | 1364517-35-0 | C_15_H_20_O_2_ | 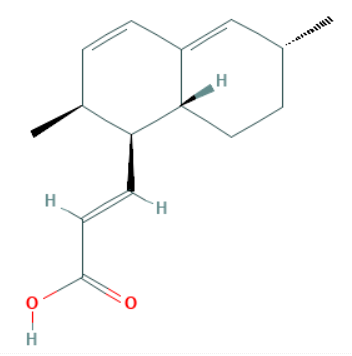 |
|  | Monascusic acid E | 1364517-37-2 | C_15_H_20_O_2_ | 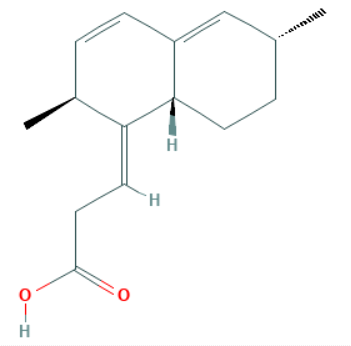 |
|  | Monascusic lactone A | 1364517-28-1 | C_15_H_20_O_2_ | 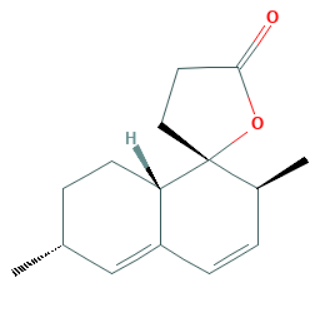 |
|  | Heptaketide | 531523-95-2 | C_15_H_24_O_2_ | 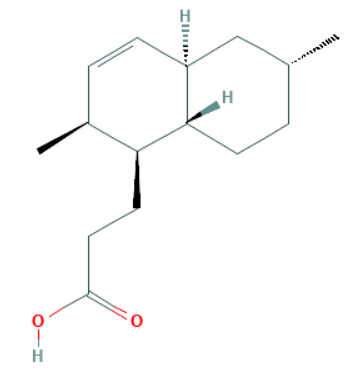 |
| Flavonoids, lignans, and coumarin | Daidzein | 486-66-8 | C_15_H_10_O_4_ | 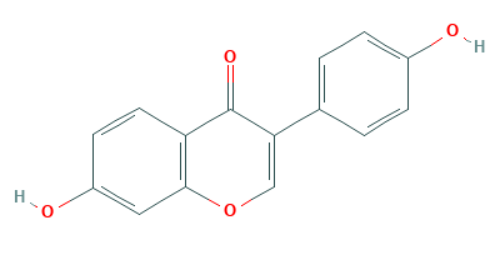 |
|  | Genistein | 446-72-0 | C_15_H_10_O_5_ | 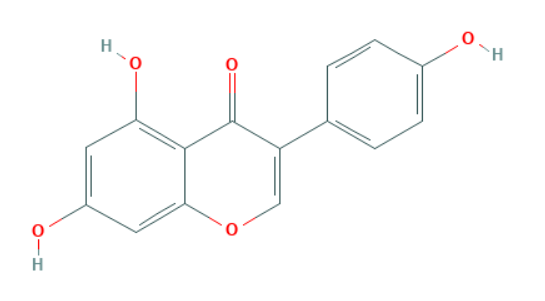 |
|  | 5,5′-Dimethoxylariciresinol | 116498-58-9 | C_22_H_28_O_8_ | 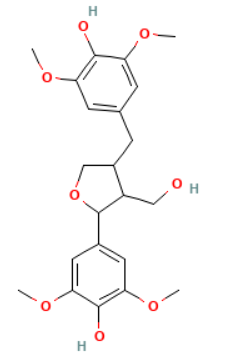 |
|  | Lariciresinol | 27003-73-2 | C_20_H_24_O_6_ | 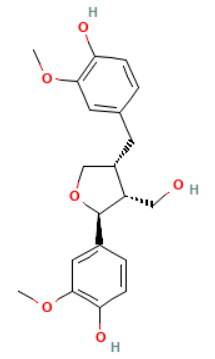 |
|  | Scopoletin | 92-61-5 | C_10_H_8_O_4_ | 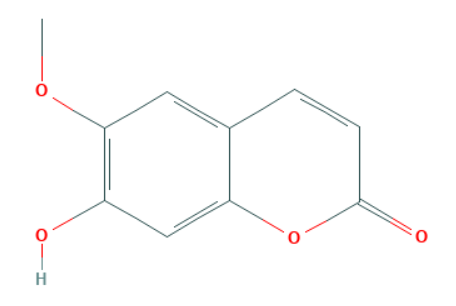 |
| Terpenoids | 3-*epi*-Betulinic acid | 472-15-1 | C_30_H_48_O_3_ | 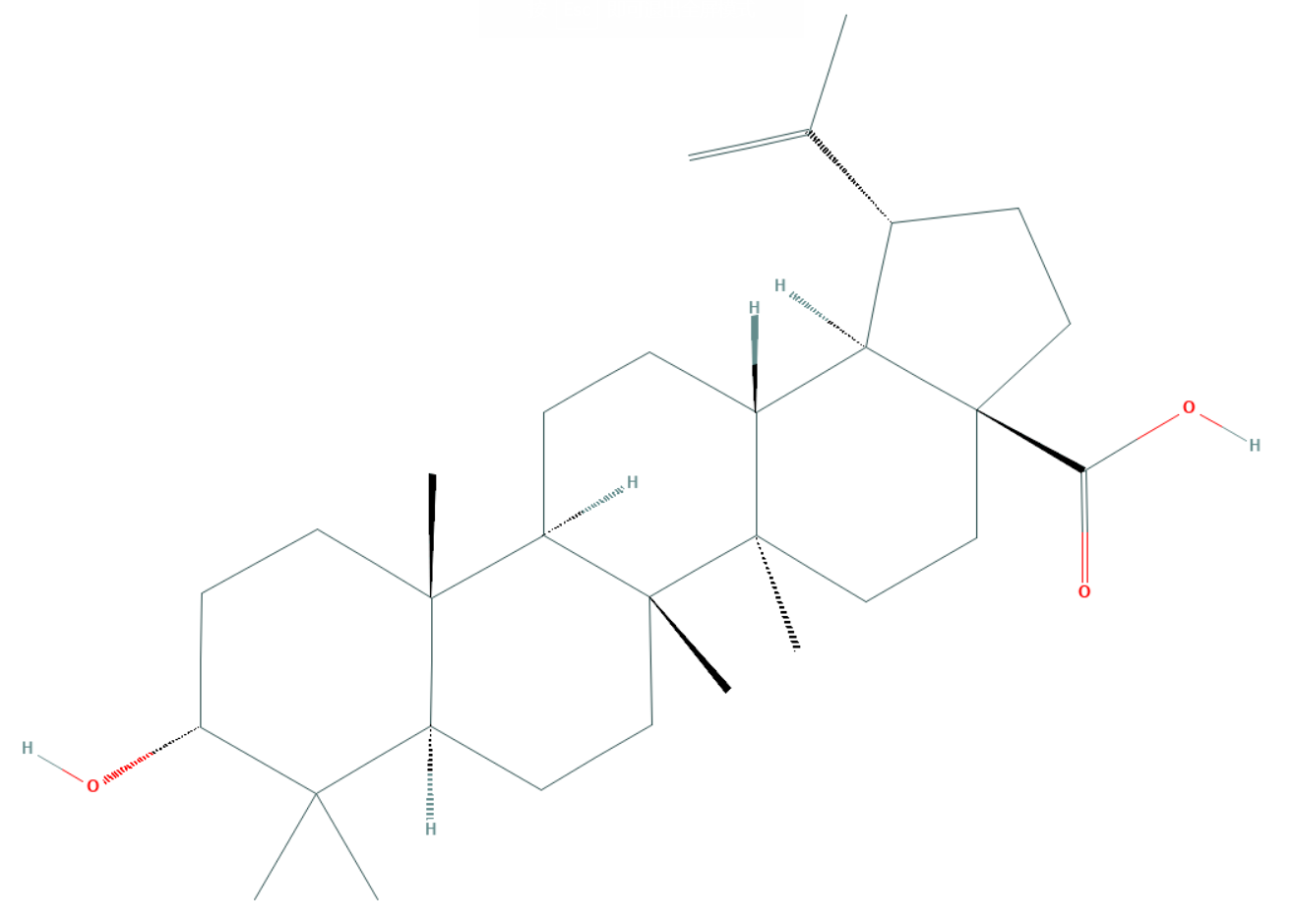 |
|  | 3-*epi*-Betulinic acid acetate | 10376-50-8 | C_32_H_50_O_4_ | 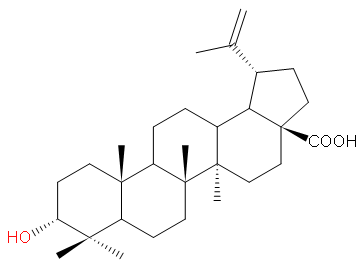 |
|  | Friedelan-3-one | 559-74-0 | C_30_H_50_O | 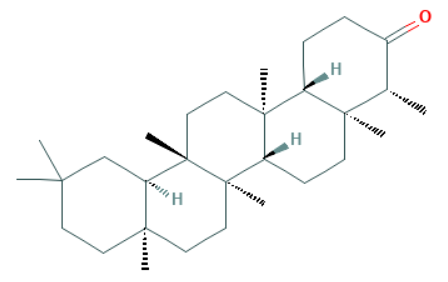 |
|  | α-Cadinol | 481-34-5 | C_15_H_26_O | 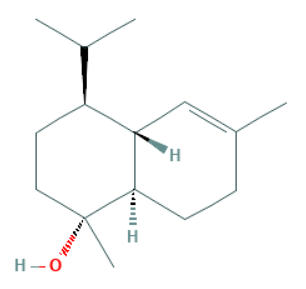 |
|  | Anticopalol | 10395-43-4 | C_20_H_34_O | 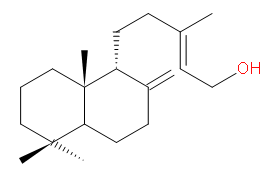 |
| Polysaccharides | EPS-1, EPS-2, EPS-3, EPS-4, EPS-5 | / | / | / |
|  | MPS-1, MPS-2, MPS-3 | / | / | / |
|  | Monascan | / | / | / |
| Other compounds | Peroxymonascuspyrone | 2227383-92-6 | C_19_H_30_O_6_ | 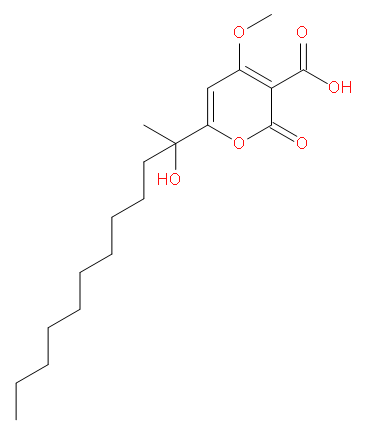 |
|  | α-Tocospiro A | 601490-40-8 | C_29_H_50_O_4_ | 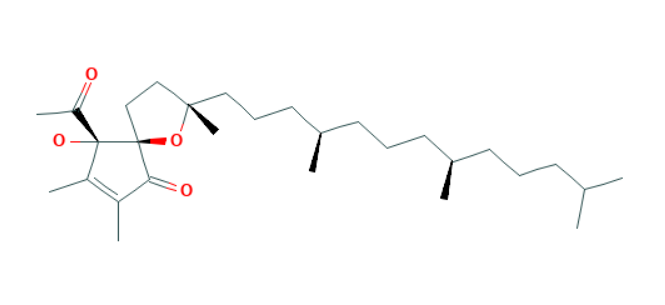 |
|  | Spathulenol | 6750-60-3 | C_15_H_24_O | 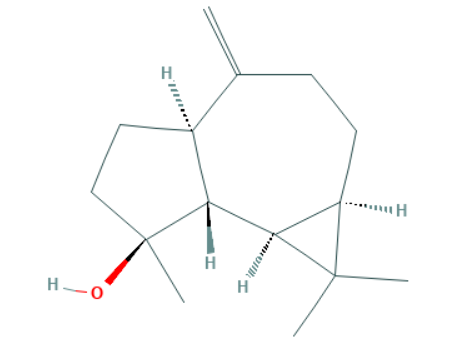 |
|  | Monascodilone | 439668-12-9 | C_15_H_12_O_4_ | 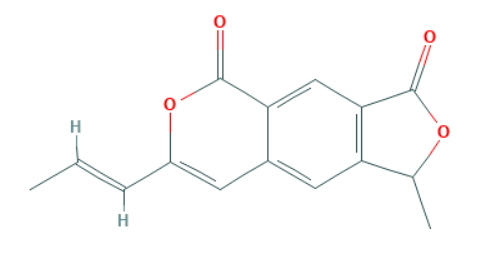 |
|  | Monascustin | 2083632-09-9 | C_10_H_18_N_2_O_3_ | 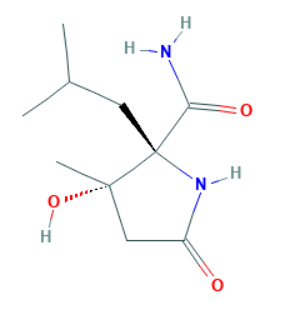 |
|  | N-*cis*-feruloylmethoxytyramine | 78510-19-7 | C_19_H_21_NO_5_ | 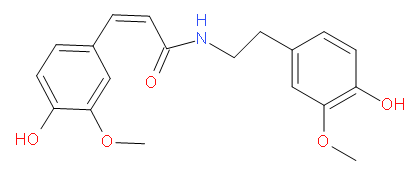 |
|  | Monaspurpurone | 1262840-98-1 | C_13_H_14_O_5_ | 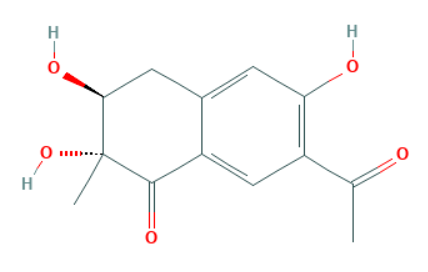 |
|  | *p*-Nitrophenol | 100-02-7 | C_6_H_5_NO_3_ | 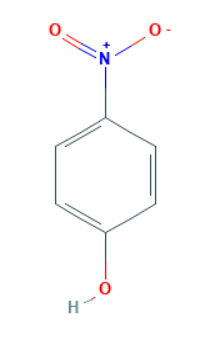 |
|  | 1-Dotriacontanol | 544-85-4 | C_32_H_66_ | 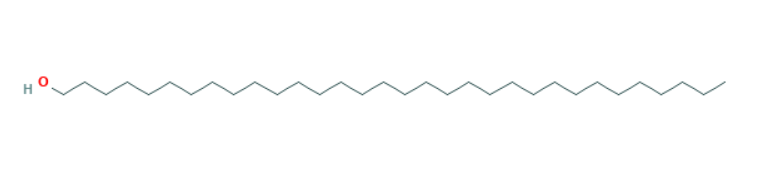 |

**Supplementary Table S4:** **Contents of chemical constituents in** ***Red yeast rice* from different manufacturers**

| Study | Formulation | Source | Species, concentration | Quality control reported (Y/N) | Chemical analysis reported (Y/N) |
| --- | --- | --- | --- | --- | --- |
| Heber 1999 | *Red yeast rice* | Pharmanex, Inc, Simi Valley, CA | Rice starch, 73.4%; Fiber, 0.8%; Protein, 5.8%; Moisture, 3-6%; Total natural pigment, <0.33%; Ash, <3%; Phosphorus (organic phosphorus 0.02%), 0.44%; Trace elements, Trace; Total HMG-CoA reductase inhibitors, 0.4% (Monacolin K, 0.2%; Monacolin K (hydroxy-acid form), 0.1%; Dihydromonacolin, <0.01%; Monacolin I, 0.03%; Monacolin II (hydroxy-acid form), <0.01%; Monacolin III, 0.02%; Monacolin IV, 0.02%; Monacolin V, 0.02%; Monacolin VI, 0.01%); Fatty acids (Saturated (palmitic and stearic), <0.5%; Mono- and polyunsaturated, <1.5%). | Y | Y; HPLC |
| Zhao 2003 | *Xuezhikang* | WBL Peking University Biotech Co., Ltd., China | Each capsule (300 mg *Red yeast rice*) contains 2.5 mg natural occurring lovastatin. | N | N |
| Lin 2005 | *Monascus purpureus Went rice* | Y & B Pharmaceuticals Co., Ltd, Taipei, Taiwan | Protein, 17.00%; Starch, 68.00%; Fat, 4.00% (Of which: Linoleic acid, 48.13%; Oleic acid, 28.78%; Palmitic acid, 18.61%; Stearic acid, 4.49%; Ergosterol, 0.30%); Fiber, 2.00%; Water , <5.00%; HMG-CoA reductase inhibitors (statins), 1.16% (Lovastatin, 0.95%; Other statins, 0.21%); r-Aminobutyric acid (GABA), 2.55%; Alkaloids (Water-soluble, 0.30%; Lipid-soluble, 0.05%); Glycosides, 0.06%; Flavonoids, 0.05%; Natural pigments, 0.01%; Ethanol extracts ≥12.00%; Water extracts ≥10.00%. | Y | Y; HPLC |
| Becker 2009 | *Red yeast rice capsule* | Sylvan Bioproducts, Kittanning, Pennsylvania | Each capsule (300 mg *Red yeast rice*) contains 0.0120mg Monacolin JA, 0.0186mg Monacolin J, 0.0080mg Monacolin XA, 0.607mg Monacolin KA, 0.0802mg Monacolin LA, 1.02mg Monacolin K (lovastatin), 0.0546mg Monacolin L, 0.0065mg Monacolin M, and 0.212mg Dihydromonacolin K. | Y | Y; HPLC |
| Yang 2009 | *Red yeast rice* | NR | NR | N | N |
| Bogsrud 2010 | *Red yeast rice* | Wearnes Biotech & Medicals Pte, Singapore | Each capsule (*Red yeast rice*) contains 1.2mg monacolin K in the lactone form (lovastatin), 0.6mg monacolin K in the hydroxyl-acid form (active lovastatin after bioconversion), and 0.6mg monacolins in the other forms. | N | N |
| Halbert 2010 | *Red yeast rice* | Sylvan Bioproducts, Kittanning, Pennsylvania | Each capsule (600 mg *Red yeast rice*) contains 1.245mg Monacolin K (lovastatin) and 0.54mg Monacolin KA. | Y | Y; HPLC |
| Karl 2012 | *Red yeast rice* | NR | 600 mg *Red yeast rice* contains 2.4mg monacolin K (lovestatin). | N | N |
| Cicero 2013 | *Red yeast rice* | Labomar s.r.l., Istrana, Treviso, Italy | Each tablet (*Red yeast rice*) contains 10 mg monacolins from *Monascus purpureus* and 10 mg coenzyme Q10. | Y | Y; HPLC |
| Verhoeven 2013 | *Red yeast rice* | NR | Each capsule (*Red yeast rice*) contains 5,025mg Monacolin K, 30mg Ubiquinone (coenzyme Q10), 20mg Procyanidins (Vitisvinifera L), and 100mg Lecithin. | Y | Y; HPLC |
| Moriarty 2014 | *Xuezhikang* | WBL Peking University Biotech Co., Ltd., China | NR | N | N |
| Cui 2015 | *Xuezhikang* | WBL Peking University Biotech Co., Ltd., China | NR | N | N |
| Wang 2015 | *Xuezhikang* | NR | NR | N | N |
| Heinz 2016 | *Red yeast rice* | MEDA Pharma, Bad Homburg, Germany | Each tablet (200mg *Red yeast rice*) contains 304.6mg Dicalcium phosphate, 253.2mg Microcrystalline cellulose, 3.0mg Monacolin K, 0.5mg Astaxanthin, 8.0mg Magnesium stearate, 8.0mg Mono- and diglycerides of fatty acids, 4.0mg Silicon dioxide, 2.0mg Coenzym Q10, and 0.2mg Pteroyl monoglutamic acid (folic acid). | Y | Y; HPLC |
| Cicero 2017 | *Red yeast rice* | NR | Each liquid stick (*Red yeast rice*) contains 5mg monacolins from *Monascus purpureus*. | N | N |

NR: no reported; Y: yes; N: no; HPLC: High performance liquid chromatography.

**Supplementary Table S5:** **Excluded** **trials with *Jadad score* less than 4.**

| **No.** | **Trial (citation format)** |
| --- | --- |
| 1 | Cicero, A., Morbini, M., Parini, A., Urso, R., Rosticci, M., Grandi, E., et al. (2016). Effect of red yeast rice combined with antioxidants on lipid pattern, hs-CRP level, and endothelial function in moderately hypercholesterolemic subjects. *Ther Clin Risk Manag*, 281. doi:10.2147/TCRM.S91817. |
| 2 | Gheith, O., Sheashaa, H., Abdelsalam, M., Shoeir, Z., and Sobh, M. (2008). Efficacy and safety of Monascus purpureus Went rice in subjects with secondary hyperlipidemia. *Clin Exp Nephrol*, 12, 189–194. doi:10.1007/s10157-008-0033-x. |
| 3 | Gheith, O., Sheashaa, H., Abdelsalam, M., Shoeir, Z., and Sobh, M. (2009). Efficacy and safety of Monascus purpureus Went rice in children and young adults with secondary hyperlipidemia: A preliminary report. *Eur J Intern Med*, 20, e57–e61. doi:10.1016/j.ejim.2008.08.012. |
| 4 | Giovanni, S., Silvia, B., Eugenio, R., Stefania, F., Romina, V., and Annunziata, L. (2013). Mediterranean Diet and Red Yeast Rice Supplementation for the Management of Hyperlipidemia in Statin-Intolerant Patients with or without Type 2 Diabetes. *Evid Based Complement Alternat Med*, 2013, 1–7. doi:10.1155/2013/743473. |
| 5 | Guardamagna, O., Abello, F., Baracco, V., Stasiowska, B., and Martino, F. (2011). The treatment of hypercholesterolemic children: Efficacy and safety of a combination of red yeast rice extract and policosanols. *Nutr Metab Cardiovasc Dis*, 21, 424–429. doi:10.1016/j.numecd.2009.10.015. |
| 6 | Hu, C.-L., Li, Y.-B., Tang, Y.-H., Chen, J.-B., Liu, J., Tang, Q.-Z., et al. (2006). Effects of Withdrawal of Xuezhikang, an Extract of Cholestin, on Lipid Profile and C-reactive Protein: A Short-Term Time Course Study in Patients with Coronary Artery Disease. *Cardiovasc Drugs Ther*, 20, 185–191. doi:10.1007/s10557-006-7947-x. |
| 7 | Huang, C.-F., Li, T.-C., Lin, C.-C., Liu, C.-S., Shih, H.-C., and Lai, M.-M. (2007). Efficacy of Monascus purpureus Went rice on lowering lipid ratios in hypercholesterolemic patients. *Eur J Cardiov Prev R*, 14, 438–440. doi:10.1097/HJR.0b013e32801da137. |
| 8 | Klimek, M., Wang, S., and Ogunkanmi, A. (2009). Safety and efficacy of red yeast rice (Monascus purpureus) as an alternative therapy for hyperlipidemia. *P T*, 34, 313–327. PMID: 19572049 |
| 9 | Li, J.-J., Hu, S.-S., Fang, C.-H., Hui, R.-T., Miao, L.-F., Yang, Y.-J., et al. (2005). Effects of Xuezhikang, an extract of cholestin, on lipid profile and C-reactive protein: a short-term time course study in patients with stable angina. *Clin Chim Acta*, 352, 217–224. doi:10.1016/j.cccn.2004.09.026. |
| 10 | Peng, D., Fong, A., and Pelt, A. van (2017). Original Research: The Effects of Red Yeast Rice Supplementation on Cholesterol Levels in Adults. *Am J Nurs*, 117, 46–54. doi:10.1097/01.NAJ.0000521973.38717.2e. |
| 11 | Ross, S. M. (2017). Red Yeast Rice: The Efficacy of Monascus Purpureus Yeast for Treatment of Hyperlipidemia a Modifiable Risk Factor of Cardiovascular Disease. *Holist Nurs Pract*, 31, 52–58. doi:10.1097/HNP.0000000000000192. |
| 12 | Wang, J., Lu, Z., Chi, J., Wang, W., Su, M., Kou, W., et al. (1997). Multicenter clinical trial of the serum lipid-lowering effects of a Monascus purpureus (red yeast) rice preparation from traditional Chinese medicine. *Curr Ther Res*, 58, 964–978. doi:10.1016/S0011-393X(97)80063-X. |
| 13 | Wang, T.-J., Lien, A. S.-Y., Chen, J.-L., Lin, C.-H., Yang, Y.-S., and Yang, S.-H. (2019). A Randomized Clinical Efficacy Trial of Red Yeast Rice (Monascus pilosus) Against Hyperlipidemia. *Am J Chin Med*, 47, 323–335. doi:10.1142/S0192415X19500150. |
| 14 | Xue, Y., Tao, L., Wu, S., Wang, G., Qian, L., Li, J., et al. (2017). Red yeast rice induces less muscle fatigue symptom than simvastatin in dyslipidemic patients: a single center randomized pilot trial. *BMC Cardiovasc Disord*, 17, 127. doi:10.1186/s12872-017-0560-z. |

**Supplementary Figure S1: Funnel plot on the sa­fety and efficacy of red yeast rice for hyperlipidemia.**

**
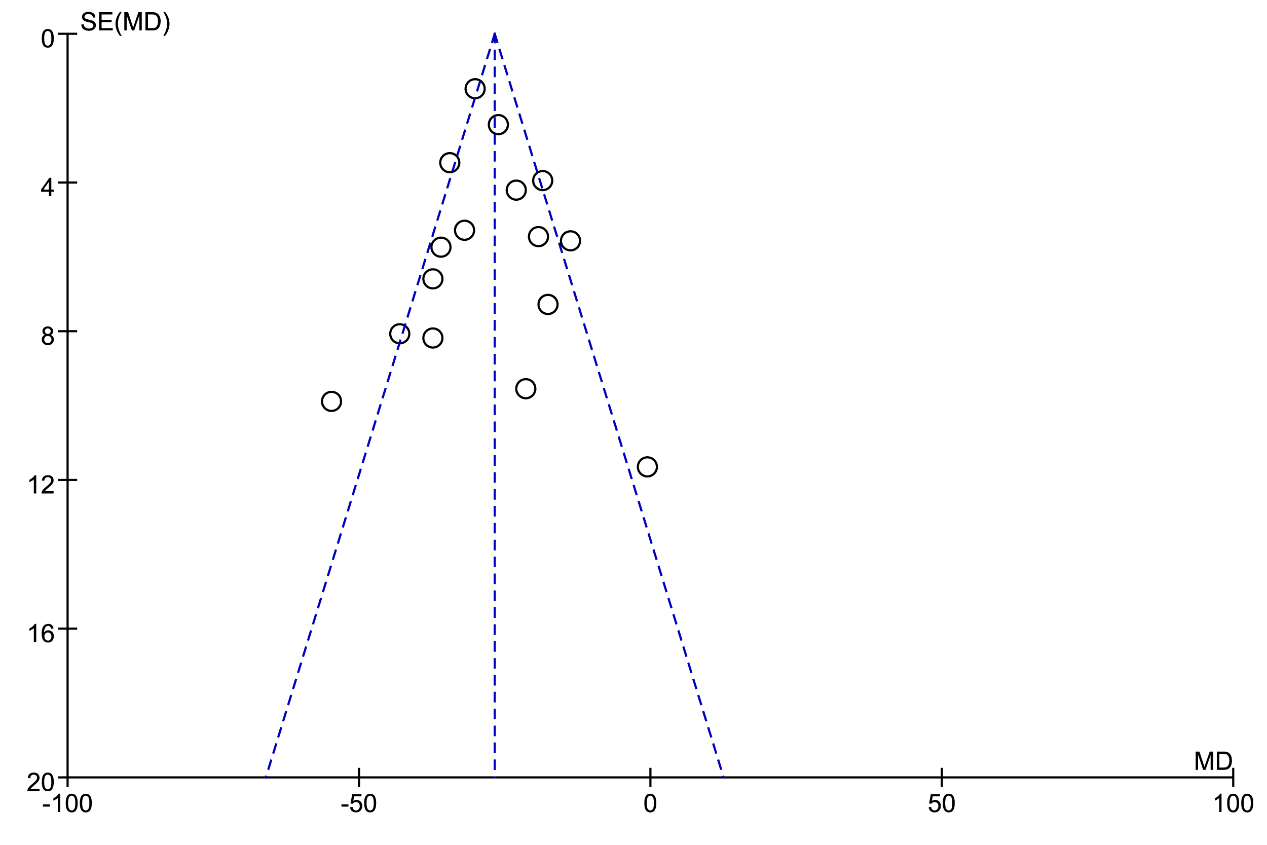
**

**Supplementary Figure S2: Forest plot of HDL-C between RYR alone and control groups.**

(RYR: red yeast rice; HDL-C: high density lipoprotein cholesterol)


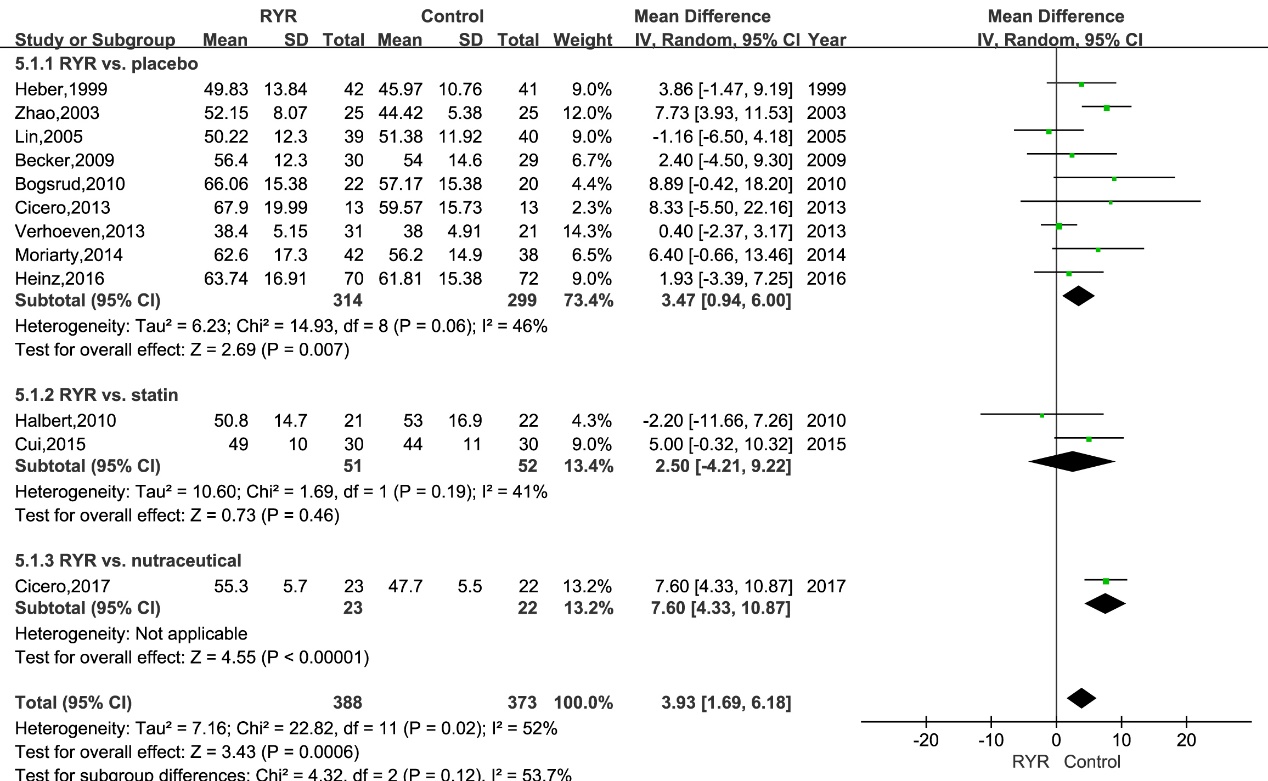


**Supplementary Figure S3: Forest plot of HDL-C between RYR in combination and control groups.**

(RYR: red yeast rice; HDL-C: high density lipoprotein cholesterol)


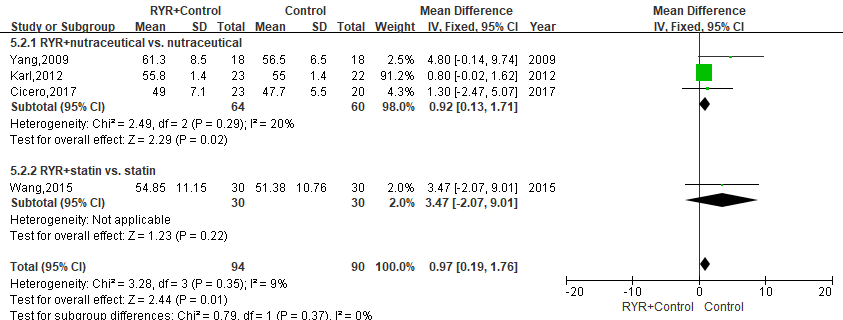


**Supplementary Figure S4. Forest plot of apoA-I between RYR and control groups.**

(RYR: red yeast rice; apoB: apolipoprotein B)


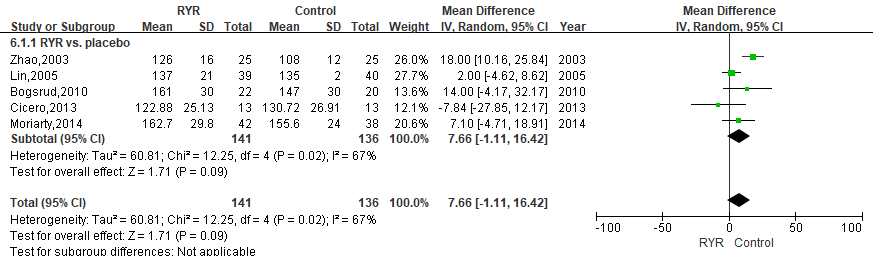


**Supplementary Figure S5: Forest plot of apoB between RYR and control groups.**

(RYR: red yeast rice; apoB: apolipoprotein B)

**
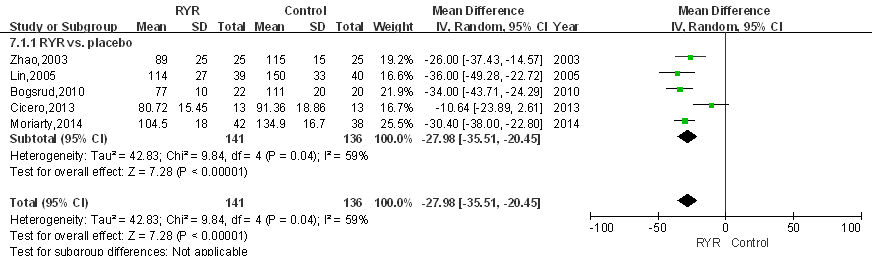
**
